# Therapeutic Implications of GIPC1 Silencing in Cancer

**DOI:** 10.1371/journal.pone.0015581

**Published:** 2010-12-30

**Authors:** Thomas W. Chittenden, Jane Pak, Renee Rubio, Hailing Cheng, Kristina Holton, Niall Prendergast, Vladimir Glinskii, Yi Cai, Aedin Culhane, Stefan Bentink, Mathew Schwede, Jessica C. Mar, Eleanor A. Howe, Martin Aryee, Razvan Sultana, Anthony A. Lanahan, Jennifer M. Taylor, Chris Holmes, William C. Hahn, Jean J. Zhao, J. Dirk Iglehart, John Quackenbush

**Affiliations:** 1 Functional Genomics and Computational Biology Group, Department of Biostatistics and Computational Biology and Department of Cancer Biology, Dana-Farber Cancer Institute, Boston, Massachusetts, United States America; 2 Department of Biostatistics, Harvard School of Public Health, Boston, Massachusetts, United States of America; 3 Department of Statistics, Oxford Centre for Gene Function, University of Oxford, Oxford, United Kingdom; 4 Department of Cancer Biology, Dana-Farber Cancer Institute, Harvard Medical School, Boston, Massachusetts, United States America; 5 Sidney Kimmel Comprehensive Cancer Center, Johns Hopkins University and Department of Biostatistics, Johns Hopkins Bloomberg School of Public Health, Baltimore, Maryland, United States America; 6 Department of Internal Medicine, Yale University School of Medicine, New Haven, Connecticut, United States America; 7 CSIRO Plant Industry, Canberra, Australia; 8 Mammalian Genetics Unit, Medical Research Council Harwell, Oxfordshire, United Kingdom; 9 Department of Medical Oncology and Center for Cancer Genome Discovery, Dana-Farber Cancer Institute, Boston, Massachusetts, United States America; 10 Broad Institute of Harvard and Massachusetts Institute of Technology, Cambridge, Massachusetts, United States America; Virginia Tech, United States of America

## Abstract

GIPC1 is a cytoplasmic scaffold protein that interacts with numerous receptor signaling complexes, and emerging evidence suggests that it plays a role in tumorigenesis. GIPC1 is highly expressed in a number of human malignancies, including breast, ovarian, gastric, and pancreatic cancers. Suppression of GIPC1 in human pancreatic cancer cells inhibits *in vivo* tumor growth in immunodeficient mice. To better understand GIPC1 function, we suppressed its expression in human breast and colorectal cancer cell lines and human mammary epithelial cells (HMECs) and assayed both gene expression and cellular phenotype. Suppression of GIPC1 promotes apoptosis in MCF-7, MDA-MD231, SKBR-3, SW480, and SW620 cells and impairs anchorage-independent colony formation of HMECs. These observations indicate GIPC1 plays an essential role in oncogenic transformation, and its expression is necessary for the survival of human breast and colorectal cancer cells. Additionally, a GIPC1 knock-down gene signature was used to interrogate publically available breast and ovarian cancer microarray datasets. This GIPC1 signature statistically correlates with a number of breast and ovarian cancer phenotypes and clinical outcomes, including patient survival. Taken together, these data indicate that GIPC1 inhibition may represent a new target for therapeutic development for the treatment of human cancers.

## Introduction

GIPC1, GIPC2 and GIPC3 comprise the human GIPC gene family, which is characterized by a single, conserved PDZ domain and GIPC homology (GH1 and GH2) domains [1]. GIPC1 is a scaffold protein involved in cell surface receptor expression, intracellular trafficking, and signal transduction. We previously showed GIPC1 plays a central role in physiologic growth factor signaling, endothelial cell regulation, and arterial branching morphogenesis in both mice and zebrafish [2], [3]. Moreover, GIPC1 interacts with and stabilizes important receptor signaling complexes, including receptor tyrosine kinases TrkA and TrkB [4], [5], VEGF co-receptor neuropilin-1 [6], FGF co-receptor syndecan-4 [7], [8], Frizzled-3 receptor [9], IGF-1 receptor [10], the TGF-beta type III receptor [11], and endoglin [12]. These receptor complex interactions reflect the role GIPC1 plays as an adaptor protein, which links multiple growth factor-supported recognition processes to intracellular signaling pathways, culminating in cell cycle regulation among other functions.

In cancer, GIPC1 was identified as an immunogenic antigen over-expressed in both breast and ovarian tumors [13], [14]. GIPC1 and GIPC2 mRNAs are expressed in OKAJIMA, TMK1, MKN45 and KATO-III human gastric cancer cells, and in various primary gastric tumors [15], [16]. GIPC1 is highly expressed in human pancreatic adenocarcinoma and plays a central role the stability of IGF-1R in pancreatic adenocarcinoma cell lines [17], [18]. Most recently, GIPC1 suppression in human pancreatic cancer cells was shown to inhibit *in vivo* pancreatic tumor growth in immunodeficient mice [19]. However, the mechanism by which GIPC1 promotes cancer growth is not well established.

To investigate the role that GIPC1 plays in cancer, we used RNAi to suppress GIPC1 expression in both breast and colorectal cancer cells and human mammary epithelial cells (HMECs). We started our study by examining alterations in global gene expression patterns after GIPC1 suppression. Our analysis indicates that GIPC1 is required for breast and colorectal cancer cell survival and plays an essential role in oncogenic transformation.

## Results

### GIPC1 silencing and gene expression patterning in MDA-MB231 human breast cancer cells

GIPC1 gene expression was knocked down in MDA-MB231 cells by transduction of short hairpin RNAs (GIPC1 KD), together with empty-vector and non-transduced controls. Following puromycin selection, seven independent biological replicates of each transduction were grown in culture, RNA was extracted, and GIPC1 knock-down was assayed using qPCR. qPCR found 85% knock-down in GIPC1 KD cells relative to non-transduced and empty-vector controls. RNAs from the independent pools were hybridized to Affymetrix Human Genome U133 Plus 2.0 GeneChips™, data were normalized using Robust Multi-Array Analysis [20] implemented in the Bioconductor *affy* package, and exploratory analysis performed with hierarchical clustering using the Bioconductor package, *made4* [21]. A strong biological effect of GIPC1 silencing was observed (Figure S1). Significance Analysis of Microarrays (SAM; q-value  = 0%) [22] was used to identify 3081 probesets (∼2271 genes) with altered expression in the GIPC1 KD cells compared to the vector control cells.

This GIPC1 KD gene list was compared to those presented by Bild et al. [23],who analyzed over-expression of five oncogenes (activated H-Ras, human E2F3, activated β-catenin, human c-Myc, and human c-Src) in primary HMECs, using *OrderedList* [24]. We found a statistically significant overlap of abnormally expressed genes in the GIPC1 KD and the activated H-Ras over-expression gene lists (P≪0.05, Figure S2).

The 3081 GIPC1 KD probesets representing 2271 genes were used in a functional enrichment analysis using Expression Analysis Systematic Explorer (EASE) [25] and nested EASE, which applies the EASE representational analysis iteratively to identify GO daughter terms that drive the significance of higher-level terms (Chittenden *et al*., submitted). EASE uses Fisher's Exact Test to identify functional classes in gene sets that are over-represented relative to the background distribution of genes assayed. Eight of 67 over-represented gene ontology (GO) terms found by EASE are shown in Table 1, among which are terms associated with cell proliferation, cell cycle, cell cycle arrest, apoptosis, cell migration, and ubiquitin-mediated protein degradation.

**Table 1:** EASE analysis of GIPC1 KD in MDA-MB231 human breast cancer cells.

Accession Type	Accession Number	Accession Term	List Hits	List Size	Pop. Hits	Pop. Size	Fisher's Exact	Corrected P Value
GO Biological Process	0016192	Cell Proliferation	277	1573	1534	12439	4.6×10^−8^	2.0×10^−3^
GO Biological Process	0007049	Cell Cycle	178	1573	936	12439	2.1×10^−9^	1.5×10^−6^
GO Biological Process	0000082	G1/S Transition of Mitotic Cycle Cell	25	1573	82	12439	1.6×10^−5^	2.1×10^−3^
GO Biological Process	0000086	G2/M Transition of Mitotic Cycle Cell	19	1573	62	12439	1.5×10^−4^	1.4×10^−2^
GO Biological Process	0007050	Cell Cycle Arrest	24	1573	98	12439	9.4×10^−4^	5.0×10^−2^
GO Biological Process	0006915	Apoptosis	150	1573	818	12439	9.0×10^−7^	2.0×10^−4^
GO Biological Process	0030334	Regulation of Cell Migration	23	1573	81	12439	1.2×10^−4^	1.2×10^−2^
GO Biological Process	0006511	Ubiquitin-Dependent Protein Catabolism	89	1573	489	12439	2.1×10^−4^	1.7×10^−2^

Table 1 presents eight over-represented EASE functional classes. The list of functional annotation classes analyzed include: GO terms for biological process, molecular function, and cellular component. *Pop Size* is the number of genes assigned to a particular annotation class. *Pop Hits* is the number of genes assigned to a particular annotation term. *List Size* indicates the number of differentially genes with assignments in each annotation class. *List Hits* is the number of differentially genes associated with each particular GO term. The *Fisher’s Exact* column lists the *p*-value from Fisher’s Exact test. The *corrected p-value column* indicates the *p-*value after correction for multiple testing by the Benjamini-Hochberg method.

Nested EASE (nEASE) is an extension of EASE that uses a second, sub-level, iterative Fisher's Exact Test to find GO subclasses that drive EASE term selection. The results, shown in Table 2, include 17 statistically enriched functional subclasses of cellular processes found by EASE. nEASE found that the EASE-significant GO biological process classes, *cell proliferation*, *protein modification*, *mitosis*, *cell growth and/or maintenance*, *cytoskeleton organization and biogenesis*, and *physiological process* are collectively driven by the biological processes *cell adhesion and integrin-mediated signaling*. Similarly, EASE-significant term *cytoplasm organization and biogenesis* was found by nEASE to be driven in part by *cytokinesis after mitosis*. nEASE found *apoptosis* significant due to GO terms associated with *regulation of actin filament* and the *JAK-STAT signaling cascade*. Altered expression was found in genes involved in both *cell migration* and *metabolism*; modifications in *ubiquitin-protein ligase activity* are due to alterations in protein binding. nEASE also indicates that GIPC1 KD alters expression of genes in the EGF, TGFβ, and WNT receptor signaling pathways.

**Table 2:** nEASE analysis of GIPC1 KD in MDA-MB231 human breast cancer cells.

nEASE Term	List Hits	List Size	Pop. Hits	Pop. Size	Fisher's Exact	Gene Enrich	nEASE pvalue Diff	nEASE Gene Enrich	% Gene Enrich	EASE Term
Cell Adhesion	27	277	104	1534	2.4×10^−2^	8.22	1.46	12.46	7.90	Cell Proliferation
Focal Adhesion Formation	4	655	10	4596	4.2×10^−2^	2.57	0.11	0.09	25.75	Cell Growth and/or Maintenance
Cell Adhesion	3	49	4	227	3.2×10^−2^	2.14	1.34	6.38	53.41	Mitosis
Cell Adhesion	14	83	37	457	2.5×10^−3^	7.28	2.44	11.52	19.67	Cytoskeleton organization and Biogenesis
Actin filament Based Movement	2	277	2	1534	3.3×10^−2^	1.64	0.87	0.17	81.94	Cell Proliferation
Negative Regulation of RHO Protein Signal Transduction	2	277	2	1534	3.3×10^−2^	1.64	0.87	0.17	81.94	Cell Proliferation
Cytokinesis after Mitosis	3	122	3	735	4.5×10^−3^	2.50	0.21	0.01	83.40	Cytoplasm Organization and Biogenesis
Establishment of Apical/Basal Cell Polarity	2	213	2	1307	2.7×10^−2^	1.67	0.67	0.31	83.70	Protein Modification
Integrin-Mediated Signaling Pathway	10	1451	39	10985	2.7×10^−2^	4.85	0.56	1.05	12.43	Physiological Process
Regulation of Actin Filament Length	4	150	4	818	1.1×10^−3^	3.26	1.84	0.60	81.66	Apoptosis
Positive Regulation of JAK-STAT Cascade	2	150	2	818	3.3×10^−2^	1.64	0.38	0.14	81.66	Apoptosis
Positive Regulation of Cell Migration	9	1054	22	7530	1.8×10^−3^	5.92	0.83	0.84	26.91	Metabolism
Ubiquitin-Protein Ligase Activity	20	1481	95	11233	2.2×10^−2^	7.50	0.42	0.99	7.86	Binding
EGF Receptor Signaling Pathway	6	118	13	705	1.2×10^−2^	3.82	0.74	0.25	29.42	Regulation of Cellular Process
TGFBeta Receptor Signaling Pathway	24	1451	96	10985	1.3×10^−3^	11.32	0.32	0.35	11.80	Physiological Process
Transforming Growth Factor Beta Receptor Activity	6	123	13	727	1.3×10^−2^	3.80	0.72	0.22	29.24	Regulation of Biological Process
WNT Receptor Signaling Pathway	17	1451	80	10985	3.0×10^−2^	6.43	0.77	2.50	8.04	Physiological Process

Table 2 presents 17 over-represented nEASE functional classes nested within enriched EASE GO terms of the upper-level EASE analysis. *Gene Erich* indicates the number of differentially expressed genes above what is expected for the nEASE *List Hits* category based on the EASE GO term enrichment. *Pvalue log diff* indicates the Fisher’s Exact Test *p*-value log difference between the same nEASE and EASE GO terms. *nEASE Gene Enrich* presents the enriched gene value based on the same EASE Gene Enrich value. *% Gene Enrich* column indicates percent gene enrichment for each nEASE GO Term based on the *Gene Enrich* value.

Collectively, these data suggest that GIPC1 is involved in cell proliferation, apoptosis, cell motility and adhesion, and ubiquitin-mediated protein degradation. These data suggest that GIPC1 plays a broad role beyond its early association with vascular regulation and that it may, in cancer, be involved in the processes associated with cell- cycle control.

### GIPC1 silencing inhibits MDA-MB231 proliferation and induces apoptosis

**Figure 1:**
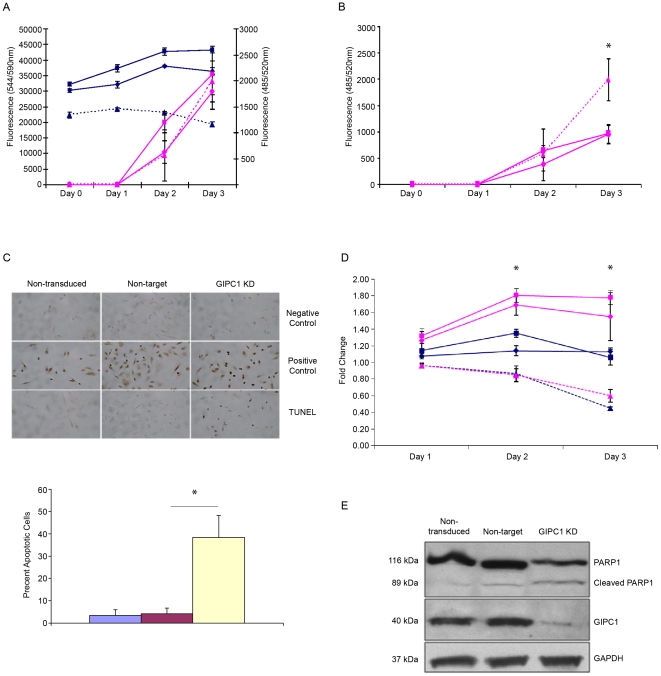
The effects of GIPC1 silencing on cell proliferation and apoptosis in MDA-MB231 human breast cancer cells. A. Assessment of cell viability (blue) and caspase 3/7 activity (pink) at 0, 24, 48, and 72 hours. Solid lines with blue boxes (non-transduced) and blue diamonds (non-target) and dashed line with blue triangles (GIPC1 KD) indicate cell viability. Pink denotes caspase 3/7 activity. B. Normalized caspase 3/7 activity. Total caspase 3/7 activity was normalized to cell viability and assessed at 0, 24, 48, and 72 hours. Normalization indicates a 2.1 fold increase in caspase 3/7 activity in GIPC1 KD cells compared to non-target cells at 72 hours. C. Tunnel assay. DNA fragmentation was assessed as % positive control. GIPC1 KD correlates with an 11.24 fold increase in apoptosis (GIPC1/Non-target; P<0.05). D. Evaluation of cell proliferation after VEGF (10 ng/ml) induction. Proliferation was evaluated at 0, 24, 48, and 72 hours. Days 1, 2, and 3 were normalized to day 0. Solid lines with blue boxes (non-transduced) and blue diamonds (non-target) and dashed line with blue triangles (GIPC1 KD) indicate cell proliferation in starvation media. Pink denotes VEGF induction. D. Evaluation of cleaved PARP1. PARP1, cleaved PARP1, GIPC1, and GAPDH expression was assessed by western blot in MDA-MB231 human breast cancer cells. Data are presented as means ± SEM. Asterisks indicate statistical significance (P≤0.05) between non-target and GIPC1 KD conditions.

Because GIPC1 KD affects processes linked to cell growth, we assessed the effects of GIPC1 depletion on MDA-MB231 cell proliferation. We determined cell viability after GIPC1 depletion by both resazurin and MTS tetrazolium reduction assays in experimental and control cells. In both instances, GIPC1 silencing causes a significant reduction in cell viability at 48 hours after seeding (Figure 1A and 1D). Since GIPC1 interacts with neuropilin-1[6], cells were treated with VEGFA (10 ng/ml) to determine whether the loss of cell viability caused by GIPC1 suppression could be overcome by a relevant mitogen. VEGFA treatment is not sufficient to prevent an approximate 40% and 60% reduction in cell viability in GIPC1 KD cells at 48 and 72 hours, respectively (Figure 1D).

GIPC1 KD also influences apoptosis. To determine the effects GIPC1 silencing on caspase 3/7 activity, the fluorometric resazurin reduction assay shown in Figure 1A was multiplexed with an Apo-ONE homogeneous caspase-3/7 assay. When caspase 3/7 activities are normalized to cell viability values, we detected a significant increase in caspase 3/7 activity in GIPC1 KD cells when compared to control cell lines at 72 hours (Figure 1B). We found similar losses of cell viability and increased caspase 3/7 activities after GIPC1 silencing in the MCF-7 and SKBR-3 human breast cancer and SW480 and SW620 human colorectal cancer cell lines (data not shown).

To determine whether the increase in caspase 3/7 activity found in MDA-MB231 cells is associated with DNA fragmentation, we performed a DeadEnd colorimetric TUNEL assay (Figure 1C). GIPC1 targeting induces DNA fragmentation; assessed as a ratio relative to control cells, the relative ratio of apoptotic cells (GIPC1 KD/non-target) is 11.24 (P<0.05). Moreover, the increase in both caspase 3/7 activity and DNA fragmentation in GIPC1-silenced cells are associated with an increased expression of cleaved PARP1 (Figure 1D). These data are consistent with the microarray findings (Tables 1 and 2), which indicate GIPC1 silencing inhibits cell proliferation and promotes apoptosis.

### GIPC1 silencing induces MDA-MB231 G2 cell-cycle arrest

**Figure 2:**
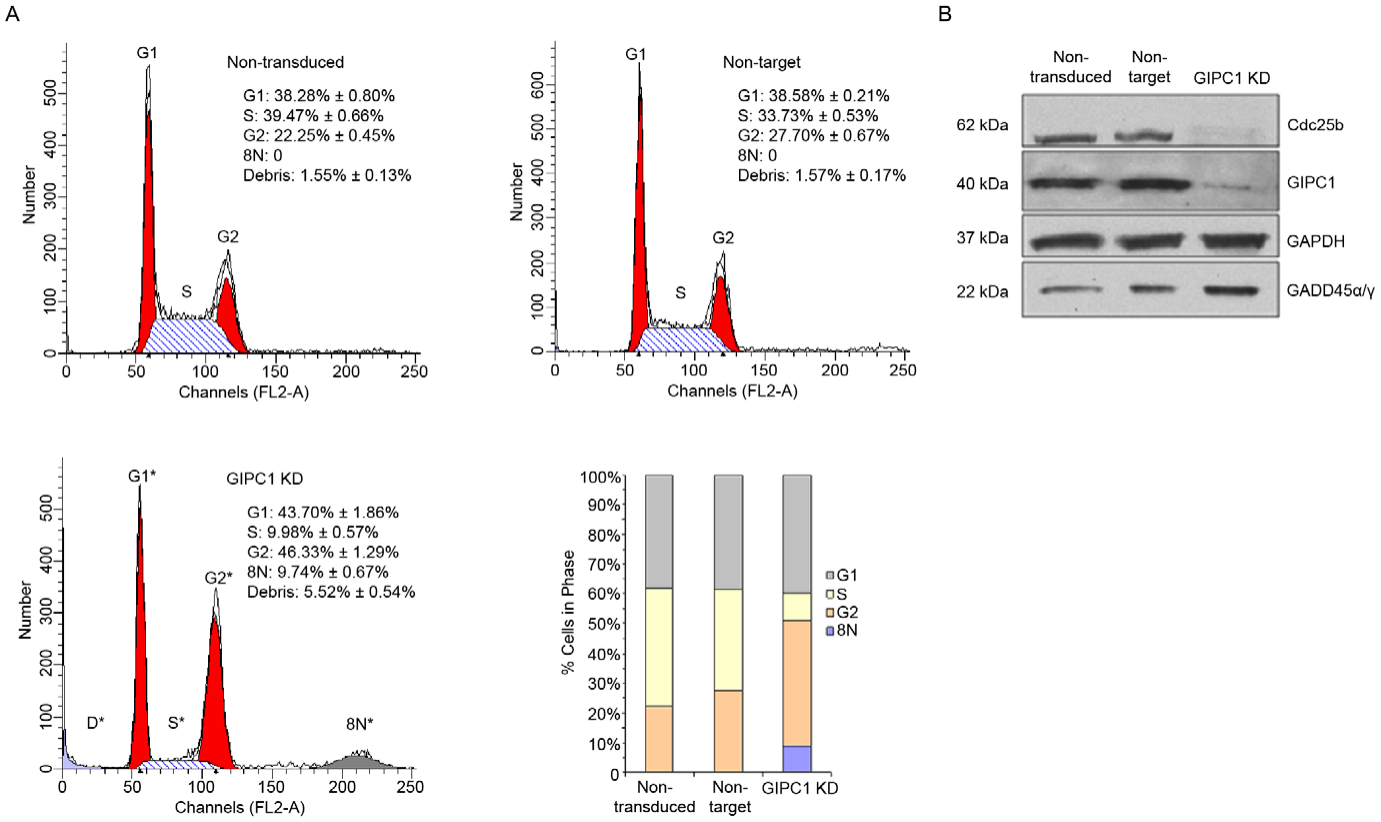
Single channel FACS analysis of GIPC1 KD MDA-MB231 human breast cancer cells. A. Single channel FACS analysis of non-transduced, non-target, and GIPC1 KD cells 14 days post-transduction. Grey is % cells in G1. Yellow indicates % cells in S phase. Orange equals % cells in G2. Blue is % cells at 8N. D indicates % debris. B. Western blot analysis of Cdc25b, GIPC1, and GADD 45 α/γ expression in non-transduced, non-target, and GIPC1 KD cells. Data are presented as means ± SEM. Asterisks indicate statistical significance (P≤0.05) between non-target and GIPC1 KD conditions.

Microarray results implicated cell cycle effects of GIPC1 KD. We performed single-channel FACS analysis of GIPC1 KD cells and the associated controls to explore the cell cycle in control and GIPC1 KD cells. GIPC1 suppression promotes subtle G1 and profound G2 arrest at day 14 post-puromycin selection of the GIPC1 shRNA transfectants (Figure 2A). These data indicate accumulation of cells in both G1 (43.70%±1.86% cells vs. 38.58%±0.21%) and G2 (46.33%±1.29% vs. 27.70%±0.67%) phases of the cell cycle in GIPC1 KD compared to non-target control cells. GIPC1 KD cells continue to traverse S phase against the prominent G2 arrest (9.98%±0.57% vs. 33.73%±0.53%). GIPC1 knockdown also results in a significant accumulation of cells in an 8N DNA peak (9.74%±0.67% vs. 0%) and in cellular debris (sub-G1; 5.52%±0.54% vs. 1.57%±0.17%). Along with the microarray findings presented in Tables 1 and 2, and compared to appropriate controls, these data suggest GIPC1 suppression promotes cell division arrest and apoptosis.

To determine causes of the abnormal cell cycle found with GIPC1 suppression, we used Western blotting to evaluate protein expression of known cell-cycle check-point regulators found differentially expressed in the microarray analysis. GIPC1 silencing induces a loss of Cdc25b and an increase in GADD45α/γ protein expression (Figure 2B). CDC25b is required for CDK1 activation and entry into mitosis; whereas, expression of the GADD45 protein family members are increased following growth arrest and DNA damage. These findings support both the microarray (Tables 1 and 2) and FACS (Figure 2A) results indicating that GIPC1 suppression predominantly promotes G2 arrest.

### GIPC1 suppression alters cell adhesion and motility

Microarray findings suggest GIPC1 is involved in adhesion and motility (Tables 1 and 2). We assessed the effects of GIPC1 silencing on cell motility and adhesion in MDA-MB231 experimental and control cells. GIPC1 silencing significantly enhanced MDA-MB231 cell adhesion at both 30 and 60 minutes after plating (Figure S3A). We used a scratch (wound) assay to assess the effects of GIPC1 KD on cell motility. GIPC1 suppression significantly decreased MDA-MB231 cell migration, either with or without eight hours of growth factor induction (Figure S3B). Microarray findings indicate GIPC1 suppression is associated with an enrichment of abnormally expressed genes within the integrin-mediated, RHO protein, JAK-STAT, EGF, Ras, TGFβ and WNT pathways (Table 2 and Figure S2). These pathways, first found in our bioinformatics analysis, regulate both cell adhesion and migration and are candidates for further investigation. Experimentally, GIPC1 depletion results in enhanced cell adhesion and reduced cell motility. GIPC1 depletion results in loss of EGF, FGF2, PDGF-BB, TGFβ1, and VEGFA induced cell motility (Figure S3B). These data suggest that GIPC1 plays a role in a number of important signal transduction pathways that impinge on both cell adhesion and motility.

### GIPC1 is required for anchorage-independent colony formation of the tHMEC-LT-st cell line

**Figure 3:**
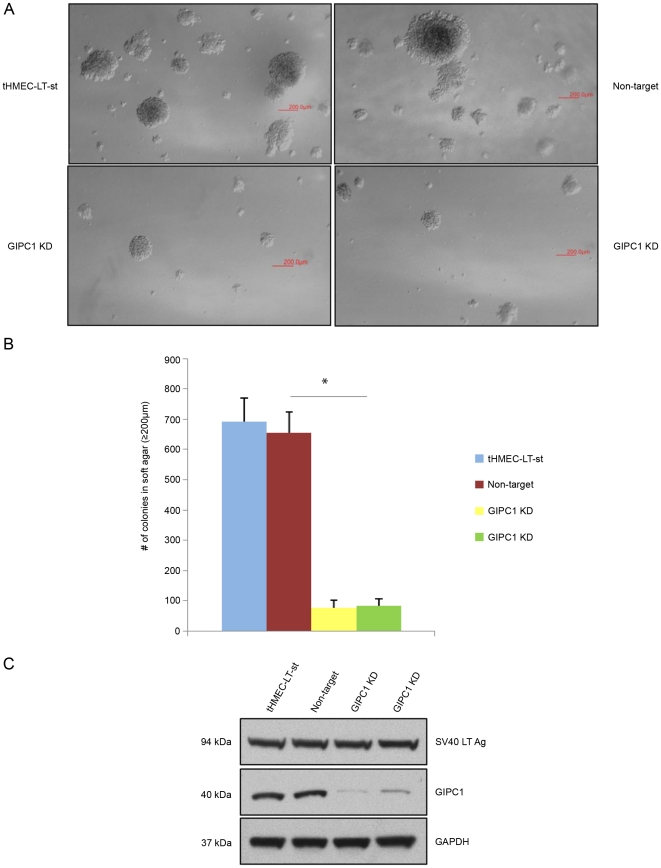
The effects of GIPC1 silencing on anchorage-independent colony formation of tHMEC-LT-st cells. A. Soft agar colony formation in nontransduced, non-target, and GIPC1 KD tHMEC-LT-st cells cells. B. Western blotting indicating the effectivness of GIPC1 silencing with two independent GIPC1 shRNA contructs: NM_005716.2-1083s1c1 and NM_005716.2-499s1c1. Data are presented as means ± SEM. Asterisks indicate statistical significance (P≤0.05) between non-target and GIPC1 KD conditions.

To assess whether GIPC1 is required for oncogenic transformation, we suppressed its expression in hTERT-immortalized HMECs transformed with SV40 Large T (LT) and Small T (st) antigens (tHMEC-LT-st) [26]. tHMEC-LT-st cells are capable of anchorage-independent growth; however, GIPC1 depletion significantly reduced the efficiency of anchorage-independent colony formation of the tHMEC-LT-st cell line when compared to control cells. As an additional control, a second GIPC1 shRNA construct was used in this set of experiments (Figure 3A). These data indicate that GIPC1 is required for anchorage-independent colony formation, a measure of oncogenic transformation of HMEC cells.

### Clinical relevance of genes regulated by GIPC1 knock-down

To further assess the assumption that GIPC1 plays a significant role in the development and progression of human cancers, 411 probesets with a fold-change ≥2 in SAM analysis of GIPC1 depleted MDA-MB231 cells compared to control cells (GIPC1 signature; Table S1) were used to interrogate two publicly available and clinically annotated breast and ovarian cancer datasets with the Bioconductor package, *globaltest* [27]. A large merged breast cancer DNA microarray dataset with 689 pretreatment samples [28], [29], [30] and an ovarian cancer dataset with 274 treated patients [31] were used for the analysis. In the Global Test multivariate analysis of the 689 breast cancer patients, the GIPC1 KD signature is associated with tumor grade (P<0.01), lymph node status (P<0.0001), and ER status (P<0.05). The GIPC1 signature is also strongly associated patient survival within the ERBB2+/ER+ (n = 42, P<0.001), luminal B (n = 146, P<0.05) and basal (n = 92, P<0.01) molecular breast cancer subtypes (Table 3). Analysis of the ovarian cancer dataset indicates that the GIPC1 signature is significantly associated with all clinical variables assessed; including patient survival and tumor stage, grade, and type (Table 3). These data support recent reports suggesting that GIPC1 plays a role in human breast and ovarian cancer etiology and progression [13], [14].

**Table 3:** Clinical relevance of the set of top 411 differentially expressed GIPC1 KD probesets (absolute fold change > 2) to human breast and ovarian cancers.

Merged Breast Cancer Dataset (N = 689)	
Clinical Variable	p-value
Recurrence-free survival	0.091
ER status	0.034
Tumor Size	0.060
LN status	8.7×10^−5^
Tumor Grade	0.0034
Age	0.065
Basal subtype	5.6×10^−22^
ERBB2+/ER- subtype	3.2×10^−6^
ERBB2+/ER+ subtype	0.016
Luminal A subtype	4.4×10^−34^
Luminal B subtype	1.9×10^−11^
Survival within basal subtype (n = 92)	0.0074
Survival within ERBB2+/ER- subtype (n = 60)	0.071
Survival within ERBB2+/ER+ subtype (n = 42)	4.3×10^−4^
Survival within luminal A subtype (n = 321)	0.13
Survival within luminal B subtype (n = 146)	0.015

Ovarian Cancer Dataset (N = 274)	
Clinical Variable	p-value
Overall survival	0.012
Tumor Stage	0.012
Tumor Grade	9.6×10^−5^
Tumor Type (malignant vs. low malignant) potential)	5.5×10^−13^

*p*-values correspond to the significance of the gene set as a predictor for the specified clinical variable, while controlling for all other variables assessed.

## Discussion

Little is known about the role of GIPC1 in tumor growth and progression. Evidence indicates it is highly expressed in a number of human malignancies, including breast, ovarian, gastric, and pancreatic cancers [13], [14]. Moreover, a recent report shows GIPC1 is required for *in vivo* pancreatic tumor growth in immunodeficient mice [19]. In this study, we used both computational and experimental approaches to examine GIPC1 in human breast and colorectal cancer cells, and in patients with breast and ovarian cancer. We found that GIPC1 is required for breast and colorectal cancer cell survival, and it plays an essential role in oncogenic transformation of human mammary epithelial cells.

Our data also show GIPC1 plays an important role in cell cycle regulation. EASE analysis of GIPC1 knockdown in MDA-MB231 cells shows enrichment of differentially expressed genes with annotated functions in G1/S and G2/M transitions, cell cycle arrest, cell proliferation, and apoptosis. nEASE seeks biological explanations for these main effects and implicates potential abnormalities in cell adhesion, integrin-mediated signaling, and regulation of the actin cytoskeleton. Additionally, nEASE found an enrichment of genes involved in cytokinesis. This finding is in agreement with a recent report indicating that activation of syndecan-4 (SDC4), a transmembrane heparan sulfate proteoglycan that interacts with GIPC1 and the FGFR1 receptor to regulate FGF2 signaling, is required for cytokinesis of MCF-7 human breast cancer cells [32]. Keller-Pinter *et al*. showed that serine179-phosphorylation and ectodomain shedding of SDC4 is maximal at G2/M. Expression of engineered mutants mimicking serine 179-phosphorylation (Ser179Glu) or phosphorylation-resistant SDC4 caused incomplete abscission and giant, multinucleated cells [32]. In addition, SDC4 regulates actin polymerization, focal adhesion formation, and cell motility through PI3K signaling.

nEASE analysis also suggests that GIPC1 is involved in six major signal transduction networks in breast cancer: integrin-mediated, RHO protein, JAK-STAT, EGF, TGFβ and WNT. These findings support previous reports indicating that GIPC1 interacts with the Frizzled-3 receptor [9], the TGF-beta type III receptor [11], and endoglin [12]. Despite the fact that our analysis did not suggest an enrichment of genes within the PI3K signal transduction network, we find that GIPC1 suppression decreases expression of a number of key genes involved in the pathway, including *SDC2, PIK3CB, PIK3D, PDK1, AKT3, CDC42BPA, CDC42EP3, RACGAP1,* and *RAC1*. Conversely, GIPC1 silencing promotes significant elevations in gene expression for *PTEN, p27^Kip1^, RHOBTB1, RHOB,* and *PAK2*. While aberrant activation of PI3K signaling is involved in the induction of oncogenic transformation, these genes work in concert to integrate mechanisms that control a number of cellular processes, including cytoskeleton regulation, cell motility and adhesion, cell proliferation, and apoptosis [26], [33].

Experimental validation of the gene expression profiling results indicates that GIPC1 silencing promotes G2 cell-cycle arrest, apoptosis, and alternations in cell adhesion and motility in MDA-MB231 human breast cancer cells. GIPC1 depletion correlates with increased caspase 3/7 activity, DNA fragmentation, upregulation of GADD45 family members, and loss of Cdc25b expression. Moreover, GIPC1 silencing correlates with marked reductions in cell viability and evaluations in caspase 3/7 activities in MCF-7 and SKBR-3 human breast cancer and SW480 and SW620 human colorectal cancer cells.

By using RNAi to deplete GIPC1 mRNA in MDA-MB-231 cells we were able to identify a wide range of genes whose expression was altered. We compared this GIPC1 signature to publicly available breast and ovarian cancer gene expression datasets for which well-annotated phenotype and outcome data were available. We found strong correlation between the GIPC1 signature and a number of important patient clinical variables.

In breast cancer, we used Global Test methodology and found recurrence-free survival was significantly associated with the GIPC1 signature only within specific molecular subtypes of the disease: patients with luminal B ER+ tumors (high-grade ER+; P = 0.015), ERBB2+/ER+ disease (P = 4.3×10^−4^), and perhaps basal-like or triple-negative cancers (P = 0.0074). Within luminal A ER+ (low-grade ER+) cases and patients with ERBB2+/ER- cancers, the GIPC1 signature was not predictive of recurrence-free survival. Therefore, the GIPC1 signature may be capable of distinguishing patient outcome within groups of high-grade breast cancers, particularly those that are ER+, and not simply distinguishing tumor grade (high vs. low) or ER status (positive versus negative). In the ovarian cancer dataset, the GIPC1 signature is statistically correlated with all clinical variables assessed: overall survival and tumor grade, type, and stage. One common feature of the correlations we found between the GIPC1 signature and clinical parameters in breast and ovarian cancer was an association with high-grade tumors that are characterized by excessive DNA damage and poor patient prognosis.

The available expression data indicate that GIPC1 is highly expressed in every human cancer and our results suggest GIPC1 is a necessary component for human cancer growth promoted by upstream growth factors and their receptors. Because GIPC1 signal transduction is activated by a wide range of cell-surface receptors and because it is also known to be essential for branching morphogenesis of arterial blood vessels, targeting GIPC1 mediated pathways is a logical therapeutic strategy for the treatment of human cancers. In particular, our data suggests targeting GIPC1 may be particularly important for estrogen receptor-positive high-grade breast cancers.

## Materials and Methods

### Cell culture

The MCF-7, MDA-MB231, and SKBR-3 human breast cancer cell lines and the SW480 and SW620 human colorectal cancer cell lines were purchased from ATCC. Human mammary epithelial cells (HMECs) were purchased from Invitrogen (#A-10565). Primary HMEC experiments were performed ≤ passage 10. *hTERT*-immortalized HMECs expressing SV40 LT and st were cultured as previously described [26]. All cell lines were cultured in 100×20 mm tissue culture dishes (Becton Dickinson #353803). MCF-7 cells were cultured at 37°C and 5% CO_2_ in EMEM (GIBCO #11095-080) supplemented with 10% heat-inactivated Fetal Bovine Serum (ATCC #30-2020) and 1% Antibiotic-Antimyotic (Invitrogen #15240-062). MDA-MB231 cells were cultured at 37°C and 0% CO_2_ in Leibovitz's L-15 media (GIBCO #11415-064) supplemented with 10% FBS and 1% Antibiotic-Antimyotic. SKBR-3 cells were cultured at 37C and 5% CO_2_ in McCoy's 5A media (GIBCO #12330-031) supplemented with 10% FBS and 1% Antibiotic-Antimyotic. SW480 and SW620 cells were cultured at 37°C and 0% CO_2_ in Leibovitz's L-15 media supplemented with 10% FBS and 1% Antibiotic-Antimyotic. Primary HMECs were cultured at 37°C and 5% CO_2_ in Medium 171PRF (phenol red-free) (GIBCO #M-171PRF-500) supplemented with mammary epithelial growth supplement (MEGS) (GIBCO #S-015-5) and 1% Antibiotic-Antimyotic. All cell lines were maintained at 60-70% confluency.

### shRNA lentiviral vector production and transduction

GIPC1(NM_005716.2-1083s1c1 and NM_005716.2-499s1c1) and empty vector shRNA plasmids as well as the Delta 8.9 and VsVg plasmids were obtained from The RNAi Facility at the Dana-Farber Cancer Institute. A scrambled, non-target shRNA plasmid was purchase from Sigma (#SHC002). cDNA versions of SV40 LT + st (LTg) were cloned into pWZL-blast as previously described[26]. All plasmids were grown in LB Broth +100 µg/µl Ampicillin (Teknova #L8105). Plasmids were purified using the Qiagen HiSpeed Plasmid Maxi Kit according to the manufacturer's instructions (Qiagen #12663), and yields were assessed using a NanoDrop spectrophotometer (Thermo Scientific #SID-10135606). HEK 293T/17 cells (ATCC # CRL-11268) were expanded to a density of 5×10^6^/100 mm cell culture dish (BD Primaria #353803) in Dulbecco's Modified Eagle's Medium (ATCC #30-2002) +10% heat-inactivated Fetal Bovine Serum (ATCC #30-2020) and incubated at 37°C, 5% CO_2_ for 24 hours. Each plate was then transfected with FuGENE 6 Transfection Reagent (Roche #11814443001), VsVG purified plasmid, Delta 8.9 purified plasmid, and either 6 µg of shRNA purified plasmid or 4 µg of purified LTg_WB_ plasmid in OptiMem media (Invitrogen #11058-021). Media was changed one day after transfection with DMEM +10% FBS media containing 1% Antibiotic-Antimyotic (Invitrogen #15240-062). Media containing the packaged virus was collected on day 2 after transfection, fresh media was added, and the viral harvest was collected again on day 3 after transfection. The packaged lentivirus was filtered with 0.45 µm filters (Corning #431220) and concentrated for 90 minutes at 16,600 g using an ultracentrifuge (Beckman #L8-70M). After resuspension, the lentivirus was titered using the QuickTiter Lentivirus Quantitation Kit (Cell Biolabs #VPK-108-HIV) per the manufacturer's instructions. For lentiviral shRNA vector transduction, growth media containing polybrene and a volume of lentivirus that equated to an MOI of 50 was added to cells and incubated overnight in cell line specific culture conditions. Next day, virus media was replaced with cell line specific growth media. At 72 hours, growth media was supplemented with either 10 µg/mL of puromycin and/or 2.5 µg/ml blasticidin. Experiments were performed 14 days after selection.

### RNA isolation, microarray hybridization and processing, and quantitative PCR

Total RNA was isolated from cell lines using the RNeasy Mini Kit (Qiagen #74106) according to the manufacturer's specifications with QIAshredder columns (Qiagen #79656) for cell pellet homogenization. Total RNA was quantified using a NanoDrop spectrophotometer (Thermo Scientific #SID-10135606). Twenty one Affymetrix GeneChip Human Genome U133 Plus 2.0 Arrays were used for this study. Seven arrays per group were used for the following MDA-MB231 cell lines: non-transduced, empty vector, and GIPC1 KD. Microarray hybridization and processing was performed using standard protocols at the Microarray Core Facility at the Dana-Farber Cancer Institute. Sample processing was performed with an Affymetrix GeneChip Fluidics Station FS450 and arrays were scanned with a GCS300 array scanner according to manufacturer's recommendations. Two-step Quantitative Real-Time PCR was performed with an Applied Biosystems 7900HT (Applied Biosystems #4329001) system. TaqMan GIPC1(Applied Biosystems #Hs00991802_m1) and 18S Endogenous Control (Applied Biosystems #4304437) assays were run at 8 replicates per sample using the Relative Quantification (Delta Ct), FAM no quench settings. Delta Cts were calculated by subtracting the average Ct of the endogenous controls from the average Ct of the amplification target. The delta-delta Ct was calculated by subtracting the average delta Ct of the empty vector samples from the average delta Ct of the target vector samples. Fold change was calculated as 2 to the power of delta-delta Ct.

### Microarray data normalization and analysis

Raw data (.cel files) were imported into R and data were normalized with RMA [20] using the Bioconductor package, *affy*. Initial exploratory data analysis was performed with hierarchical clustering analysis (average-linkage and metric 1 - Pearson correlation coefficient distance) using the Bioconductor package, *made4* [21]. Two class unpaired Significance Analysis of Microarrays (SAM;[22] with a 0% false discovery rate (q-value) was used to determine differential gene expression between empty vector control and GIPC1 KD MDA-MB231 cells.

The nested Expression Analysis Systematic Explorer (nEASE) algorithm (Chittenden *et al*., submitted) which is implemented along with SAM in the TM4 Multiple Experiment Viewer (MeV)[34], was then used to determine iterative sub-level, biological classifications for biological process, molecular function, and cellular component gene ontology (GO) terms [35]. To assess the likelihood that GO terms arise at a greater frequency than what would be expected by chance, nEASE generates a P-value from gene tallies associated with statistically enriched EASE [25] GO terms that are corrected for multiple hypothesis testing by the Benjamini-Hochberg method [36]. The gene counts associated with each enriched EASE GO term are used as background distributions for the purposes of deriving iterative sub-level nEASE hypergeometric distributions via the Fisher's Exact Test for each individual EASE GO term.

### Analysis of clinical breast and ovarian cancer public datasets

The clinical relevance of the GIPC1 KD signature (n = 411 probesets) was evaluated in publicly available breast and ovarian cancer gene expression data which were downloaded from the *Gene Expression Omnibus* database at NCBI. After excluding patients with missing clinical data, the breast cancer dataset contained 689 gene expression profiles that were obtained by merging the datasets GSE6532 [30], GSE4922 [29], and GSE7390 [28]. The ovarian cancer dataset contained 274 gene expression profiles from GSE9891 [31].

Association between gene expression of the GIPC1 KD signature and each clinical variable in the breast and ovarian cancer datasets were evaluated using globaltest [27]. Resulting P-values were adjusted for multiple testing using the Hommel approach within the “p.adjust” function of the *limma* package of Bioconductor. Ovarian and breast cancer data were treated as two independent groups of tests in multiple testing corrections. In order to relate the gene signature to specific clinical variables and avoid confounding with other correlated clinical variables, tests of a variable of interest were done while controlling all other clinical variables in the model.

Breast clinical variables included molecular subtype, lymph node status, estrogen receptor status, size, age, grade, and recurrence-free survival. Grade was treated as an ordered categorical variable. Ovarian clinical variables were size, grade, type (malignant versus. low malignant potential) and overall survival.

### Analysis of merged MDA-MB231 GIPC1 KD and HMEC oncogene signature dataset

An HMEC oncogene signature dataset, GSE3151 [23]was merged with the MDA-MB231 GIPC1 KD dataset. The merged dataset was normalized with RMA [20] using the Bioconductor package, *affy*. A meta-analysis was performed with the Bioconductor R package, *OrderedList* with default parameters [24], to determine the degree of overlap in differential gene expression between the GIPC1 KD MDA-MB231 cell line and each of the five HMEC oncogene expression cell lines (H-Ras, E2F3, β-catenin, c-Myc, c-Src).

### Western blotting

Whole cell lysates were prepared and then separated by SDS-PAGE in the following manner: cells were washed in cold PBS and lysed in cold RIPA buffer (Boston BioProducts #BP-115) containing both protease (Roche #11-873-580-001) and phosphatase (Sigma #P2850-5mL and #P5726-5mL) inhibitors. Protein concentrations were determined with a BCA protein assay kit (Pierce #23225) according to manufacturer's recommendations. Protein samples were subjected to 10% SDS-PAGE and then transferred onto PVDF membranes (Bio-RAD #162-0239). Membranes were blocked for 1 hour at room temperature in 5% non-fat milk/TBST (0.1% Tween20), and then incubated overnight at 4°C according to manufacturer's recommendations with the following antibodies: cdc25B (Cell Signaling #9525), GIPC1 (Santa Cruz Biotechnology #sc-9648), GAPDH (Santa Cruz Biotechnology #sc-32233), GADD 45α/γ (Cell Signaling #3518), and PARP (Cell Signaling #9542). Immunoblots were incubated with primary antibody specific, horseradish peroxidase-conjugated secondary antibodies from Santa Cruz Biotechnology and Cell Signaling and then developed with SuperSignal West Pico Chemiluminescent Substrate (Pierce #34078). Densitometry was performed with Scion Image software.

### Fluorescence-activated cell sorting analysis

DNA content and cell cycle progression was evaluated in GIPC1 KD, non-target, and non-transduced MDA-MB231 cell lines by a standard BrdU and propidium iodide (PI) double staining method at the Flow Cytometry Core Laboratory of the Dana-Farber Cancer Institute. Samples were analyzed using a FACScan flow cytometer analyzer and CellQuest software (Becton Dickinson). The FITC Mouse Anti- BrdU Set (anti-BrdU-FITC: 51-33284X and irrelevant IgG-FITC: 51-35404X-2) was purchased from BD Pharmingen.

### Soft agar oncogenic transformation assay

Anchorage-independent growth assays were performed as previously described[26]. Briefly, 5×10^4^ experimental and control tHMEC-LT-st cells were seeded in 60 mm plates with a bottom layer of 0.6% Bacto agar in DMEM and a top layer of 0.3% Bacto agar containing MEGM. Fresh MEGM (0.5 ml) was added after 1.5 weeks and colonies were scored after 3 weeks. Only those colonies ≥0.2 mm in diameter were counted. Colonies were imaged with a VistaVision inverted microscope and a XLI-Cap USB2.0 high resolution camera. At least two independent assays were performed in triplicate.

### Cell proliferation/viability and apoptosis assays

Anchorage-dependent growth assays were performed with a CellTiter 96 AQ_ueous_ One Solution Cell Proliferation Assay (Promega # G3582) and a CellTiter-Blue Cell Viability (Promega # G8082) assays. The CellTiter 96 AQ_ueous_ One Solution Cell Proliferation Assay was used to assess cell proliferation for 72 hours in non-transduced, non-target, and GIPC1 KD MDA-MB231 human breast cancer cell lines. Cells were seeded in triplicate at a density of 5×10^3^ in 100 µL of L-15 normal growth media/well in 96-well tissue culture plates and allowed to attach for 2 hours at 37°C, 0% CO_2_. Cells were serum starved in L-15 starvation media with 0.5% FBS for 24 hours. Media was then replaced with 100 µL of L-15 starvation media supplement with either VEGF (10 ng/ml) or 0.1% BSA/PBS. Immediately after media replacement, 20 µl of Cell Titer 96 A_Queous_ One Solution Reagent (Promega # G358A) was added to day 0 wells and plates were then incubated for 2 hours at 37°C, 0% CO_2_. Absorbance was recorded at 492 nm using a standard ELISA 96-well plate reader (Bio-Rad MPM III 1.133). This procedure was repeated every 24 hours for 72 hours to assess cell proliferation.

To assess caspase 3/7 activity, CellTiter-Blue Cell Viability (Promega # G8082) and Apo-ONE® Homogeneous Caspase-3/7 (Promega # G7792) assays were multiplexed according to manufacturer's recommendations. With the exception of growth factor induction, experimental and control cells were cultured as described above. On day 0, 20 µl/well of CellTiter-Blue Reagent was added to each well and incubated for 2 hours in normal L-15 growth media at 37°C, 0% CO_2_. CellTiter-Blue fluorescence (cell viability) was then recorded at 544_ex_/590_em_ with a standard 96-well fluorescent plate reader (BMG LabTech, FluoStar Optima). An equal volume of 1∶100 Apo-ONE Reagent (caspase substrate Z-DEVD-R110: buffer) was then added to Day 0 wells and incubated for 30 mins at room temperature on shaker. Apo-ONE fluorescence (caspase 3/7 activity) was recorded at 485_ex_/520_em_. This procedure was repeated every 24 hours for 72 hours to assess caspase 3/7 activity. Caspase 3/7 activity was normalized to GIPC1 KD cell viability. Apoptosis was evaluated with a DeadEnd Colorimetric TUNEL system (Promega #G7130) according to manufacturer's recommendations. Briefly, non-transduced, non-target, and GIPC1 KD MDA-MB231 cell lines were cultured in normal L-15 growth media and MDA-MB231 culture conditions on Poly-Prep microscope slides (Sigma #PO425). Cells were fixed in 4% paraformaldehyde and permeabilized with 0.2% Triton X-100. Positive controls were treated with DNase I buffer containing 10 unit/ml of DNase I (Promega #M6101). Experimental and positive controls cells were treated with rTdT reaction mix containing equilibration buffer, biotinylated nucleotide mix, and rTdT enzyme. For the negative control rTdT reaction mix, rTdT enzyme was replaced with deionized water. The rTdT reactions were terminated with 20X SSC and endogenous peroxidase activity was blocked with 0.3% hydrogen peroxide. Slides were treated with Streptavidin HRP solution, 1∶500 in PBS. Slides were then developed with DAB solution for 10 mins. Slides were mounted with Permount Mounting Media (Fisher # SP15-100) and imaged at 10X with an Olympus BX41 light microscope and MicroPlubisher Color CCD digital camera. DNA fragmentation was assessed as percent positive control.

### Adhesion assay

GIPC1, non-target, and non-transduced MDA-MB231 cell lines were plated in 12-well tissue-culture plates in triplicate at 5×10^4^ cells/well and allowed to attach for either 30 or 60 mins. Adherent cells were fixed with 3.7% PFA in PBS and then stained with Coomassie blue. Wells were imaged with a VistaVision inverted microscope and a XLI-Cap USB2.0 high resolution camera. Cell adhesion was determined relative to total cells plated.

### Wound (scratch) migration assay

The bottoms of 6-well tissue-culture plates were gridded for accurate measurement. 2×10^5^ GIPC1, non-target, and non-transduced MDA-MB231 cell lines were plated/well in triplicate. Cells were allowed to reach confluence and then serum starved in 0.5% FBS L-15 media for 24 hours. Scratches in cell lawns were made with a P20 pipette tip at ∼1 mm on each side of each gridline. Media was then aspirated and 0.5% FBS L-15 control media was added to each control well. 0.5% FBS L-15 control media was supplemented with one of the following growth factors and added to growth factor-specific experimental wells: EGF (50 ng/ml); FGF2 (10 ng/ml); PDGF-BB (50 ng/ml); TGFB1 (5 ng/ml); or VEGF (10 ng/ml). Wells were imaged with a VistaVision inverted microscope and a XLI-Cap USB2.0 high resolution camera at zero and eight hours post-wounding. Total migration distance was determined with XLI-Cap measurement/capture software.

### Statistical analysis of cell assays

Data are presented as means ± SEM. Results from a minimum of three independent experiments with six replicates per condition were performed on separate cell passages for each assay. Data were analyzed by repeated measures ANOVA. If analysis showed a significant difference (p≤0.05) among groups, the Student-Newman-Keuls (SNK) test for multiple comparisons of means was performed to identify the conditions that were different from one another. Asterisks indicate statistical significance (p≤0.05) between non-target and GIPC1 KD conditions.

## Supporting Information

Figure S1 **Hierarchical clustering and presentation of RMA normalized microarray data.** GI(blue): GIPC1 KD MDA-MB231 cells; PL(green): Empty vector MDA-MB231 cells control cells; CO(red): non-transduced MDA-MB231 control cells.
(TIF)

Figure S2 **Gene list overlap.** Figure S1 shows the statistically assessed overlap of differentially expressed genes between MDA-MB231 GIPC1 KD and HMEC H-Ras, E2F3, β-CAT, c-MYC, and c-SRC overexpression experiments.
(TIF)

Figure S3 **The effects of GIPC1 silencing on cell adhesion and cell motility in MDA-MB231 human breast cancer cells.** A. Cell adhesion assay: 5×10^4^ cells were plated/well in 12 well tissue culture plates. Cell adhesion was evaluated relative to total cells plated at 30 and 60 minutes after seeding at 20× magnification with an inverted microscope. B. Scratch (wound) Assay: 1×10^5^ cells were plated/well in 6 well tissue culture plates. Cells were allowed to reach confluence and then serum starved for 24 hours. A wound (scratch) was made with a P20 pipette tip. Cells were stimulated with EGF, FGF2, PDGF-BB, TGFβ1, or VEGFA and imaged at 10× magnification with an inverted microscope. Eight hours later wells were reimaged and total migration distance was assessed according to gap closure. Blue: non-transduced; red: non-target; yellow: GIPC1 KD.
(TIF)

Table S1 The set of the top 411 SAM probesets (0% FDR; fold change ≥ 2) assessed for clinical relevance to human breast and ovarian cancers with the globaltest Bioconductor R package.
(DOC)

## References

[1] Katoh M (2002). GIPC gene family (Review).. Int J Mol Med.

[2] Chittenden TW, Claes F, Lanahan AA, Autiero M, Palac RT (2006). Selective regulation of arterial branching morphogenesis by synectin.. Dev Cell.

[3] Lanahan AA, Chittenden TW, Mulvihill E, Smith K, Schwartz S (2006). Synectin-dependent gene expression in endothelial cells.. Physiol Genomics.

[4] Kato H, Ohno K, Hashimoto K, Sato K (2004). Synectin in the nervous system: expression pattern and potential as a binding partner of neurotrophin receptors.. FEBS Lett.

[5] Lou X, Yano H, Lee F, Chao MV, Farquhar MG (2001). GIPC and GAIP form a complex with TrkA: a putative link between G protein and receptor tyrosine kinase pathways.. Mol Biol Cell.

[6] Cai H, Reed RR (1999). Cloning and characterization of neuropilin-1-interacting protein: a PSD-95/Dlg/ZO-1 domain-containing protein that interacts with the cytoplasmic domain of neuropilin-1.. J Neurosci.

[7] Gao Y, Li M, Chen W, Simons M (2000). Synectin, syndecan-4 cytoplasmic domain binding PDZ protein, inhibits cell migration.. J Cell Physiol.

[8] Tkachenko E, Rhodes JM, Simons M (2005). Syndecans: new kids on the signaling block.. Circ Res.

[9] Tan C, Deardorff MA, Saint-Jeannet JP, Yang J, Arzoumanian A (2001). Kermit, a frizzled interacting protein, regulates frizzled 3 signaling in neural crest development.. Development.

[10] Ligensa T, Krauss S, Demuth D, Schumacher R, Camonis J (2001). A PDZ domain protein interacts with the C-terminal tail of the insulin-like growth factor-1 receptor but not with the insulin receptor.. J Biol Chem.

[11] Blobe GC, Liu X, Fang SJ, How T, Lodish HF (2001). A novel mechanism for regulating transforming growth factor beta (TGF-beta) signaling. Functional modulation of type III TGF-beta receptor expression through interaction with the PDZ domain protein, GIPC.. J Biol Chem.

[12] Finger EC, Lee NY, You HJ, Blobe GC (2008). Endocytosis of the type III transforming growth factor-beta (TGF-beta) receptor through the clathrin-independent/lipid raft pathway regulates TGF-beta signaling and receptor down-regulation.. J Biol Chem.

[13] Rudchenko S, Scanlan M, Kalantarov G, Yavelsky V, Levy C (2008). A human monoclonal autoantibody to breast cancer identifies the PDZ domain containing protein GIPC1 as a novel breast cancer-associated antigen.. BMC Cancer.

[14] Yavelsky V, Rohkin S, Shaco-Levy R, Tzikinovsky A, Amir T (2008). Native human autoantibodies targeting GIPC1 identify differential expression in malignant tumors of the breast and ovary.. BMC Cancer.

[15] Kirikoshi H, Katoh M (2002). Up-regulation of GIPC2 in human gastric cancer.. Int J Oncol.

[16] Kirikoshi H, Katoh M (2002). Expression of human GIPC1 in normal tissues, cancer cell lines, and primary tumors.. Int J Mol Med.

[17] Muders MH, Baretton GB, Aust DE, Dutta SK, Wang E (2007). [GIPC: a new target for therapy in pancreatic adenocarcinoma?].. Verh Dtsch Ges Pathol.

[18] Muders MH, Dutta SK, Wang L, Lau JS, Bhattacharya R (2006). Expression and regulatory role of GAIP-interacting protein GIPC in pancreatic adenocarcinoma.. Cancer Res.

[19] Muders MH, Vohra PK, Dutta SK, Wang E, Ikeda Y (2009). Targeting GIPC/synectin in pancreatic cancer inhibits tumor growth.. Clin Cancer Res.

[20] Irizarry RA, Hobbs B, Collin F, Beazer-Barclay YD, Antonellis KJ (2003). Exploration, normalization, and summaries of high density oligonucleotide array probe level data.. Biostatistics.

[21] Culhane AC, Thioulouse J, Perriere G, Higgins DG (2005). MADE4: an R package for multivariate analysis of gene expression data.. Bioinformatics.

[22] Tusher VG, Tibshirani R, Chu G (2001). Significance analysis of microarrays applied to the ionizing radiation response.. Proc Natl Acad Sci U S A.

[23] Bild AH, Yao G, Chang JT, Wang Q, Potti A (2006). Oncogenic pathway signatures in human cancers as a guide to targeted therapies.. Nature.

[24] Lottaz C, Yang X, Scheid S, Spang R (2006). OrderedList–a bioconductor package for detecting similarity in ordered gene lists.. Bioinformatics.

[25] Hosack DA, Dennis G, Sherman BT, Lane HC, Lempicki RA (2003). Identifying biological themes within lists of genes with EASE.. Genome Biol.

[26] Zhao JJ, Gjoerup OV, Subramanian RR, Cheng Y, Chen W (2003). Human mammary epithelial cell transformation through the activation of phosphatidylinositol 3-kinase.. Cancer Cell.

[27] Goeman JJ, Oosting J, Cleton-Jansen AM, Anninga JK, van Houwelingen HC (2005). Testing association of a pathway with survival using gene expression data.. Bioinformatics.

[28] Desmedt C, Piette F, Loi S, Wang Y, Lallemand F (2007). Strong time dependence of the 76-gene prognostic signature for node-negative breast cancer patients in the TRANSBIG multicenter independent validation series.. Clin Cancer Res.

[29] Ivshina AV, George J, Senko O, Mow B, Putti TC (2006). Genetic reclassification of histologic grade delineates new clinical subtypes of breast cancer.. Cancer Res.

[30] Loi S, Haibe-Kains B, Desmedt C, Lallemand F, Tutt AM (2007). Definition of clinically distinct molecular subtypes in estrogen receptor-positive breast carcinomas through genomic grade.. J Clin Oncol.

[31] Tothill RW, Tinker AV, George J, Brown R, Fox SB (2008). Novel molecular subtypes of serous and endometrioid ovarian cancer linked to clinical outcome.. Clin Cancer Res.

[32] Keller-Pinter A, Bottka S, Timar J, Kulka J, Katona R (2010). Syndecan-4 promotes cytokinesis in a phosphorylation-dependent manner.. Cell Mol Life Sci.

[33] Chu IM, Hengst L, Slingerland JM (2008). The Cdk inhibitor p27 in human cancer: prognostic potential and relevance to anticancer therapy.. Nat Rev Cancer.

[34] Saeed AI, Bhagabati NK, Braisted JC, Liang W, Sharov V (2006). TM4 microarray software suite.. Methods Enzymol.

[35] Ashburner M, Ball CA, Blake JA, Botstein D, Butler H (2000). Gene ontology: tool for the unification of biology. The Gene Ontology Consortium.. Nat Genet.

[36] Benjamini Y, Hochberg Y (1995). Controlling the False Discovery Rate: A Practical and Powerful Approach to Multiple Testing.. Journal of the Royal Statistical Society Series B (Methodological).

