# Supplementary figures and images for: Therapeutic Implications of GIPC1 Silencing in Cancer
Source: PLoS One. 2010 Dec 30;5(12):e15581. doi: 10.1371/journal.pone.0015581 (PMC3012716; doi:10.1371/journal.pone.0015581)

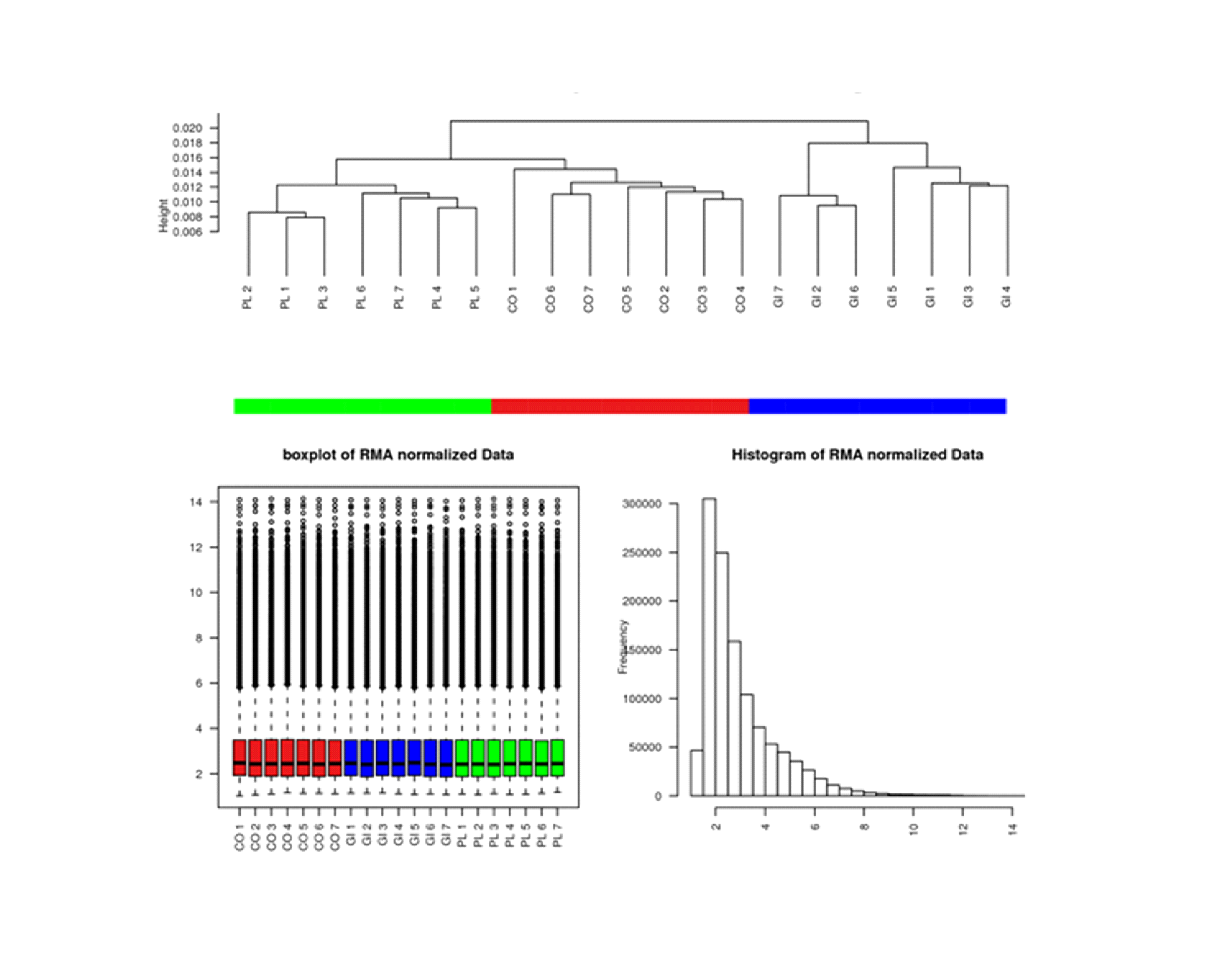

Supplement: Figure S1 — Hierarchical clustering and presentation of RMA normalized microarray data. GI(blue): GIPC1 KD MDA-MB231 cells; PL(green): Empty vector MDA-MB231 cells control cells; CO(red): non-transduced MDA-MB231 control cells. (TIF) [file pone.0015581.s001.tif]

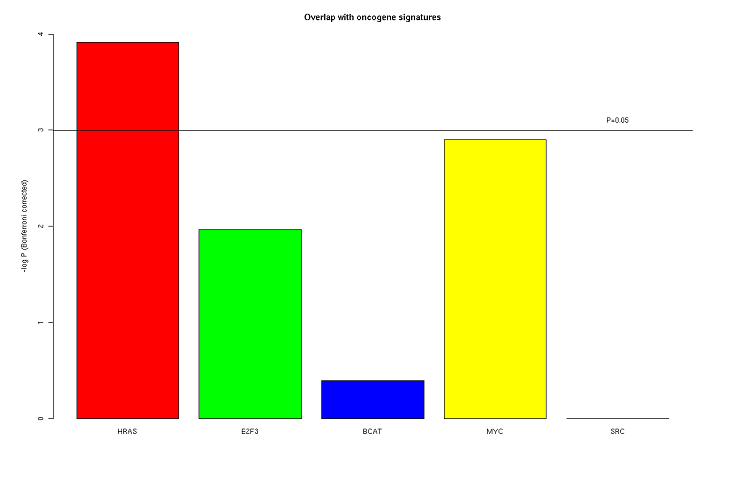

Supplement: Figure S2 — Gene list overlap. Figure S1 shows the statistically assessed overlap of differentially expressed genes between MDA-MB231 GIPC1 KD and HMEC H-Ras, E2F3, β-CAT, c-MYC, and c-SRC overexpression experiments. (TIF) [file pone.0015581.s002.tif]

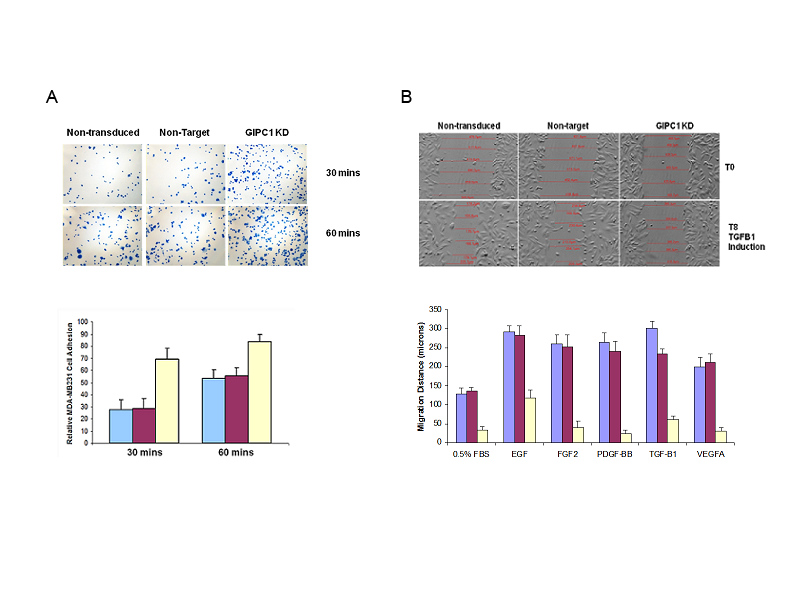

Supplement: Figure S3 — The effects of GIPC1 silencing on cell adhesion and cell motility in MDA-MB231 human breast cancer cells. A. Cell adhesion assay: 5×10^4^ cells were plated/well in 12 well tissue culture plates. Cell adhesion was evaluated relative to total cells plated at 30 and 60 minutes after seeding at 20× magnification with an inverted microscope. B. Scratch (wound) Assay: 1×10^5^ cells were plated/well in 6 well tissue culture plates. Cells were allowed to reach confluence and then serum starved for 24 hours. A wound (scratch) was made with a P20 pipette tip. Cells were stimulated with EGF, FGF2, PDGF-BB, TGFβ1, or VEGFA and imaged at 10× magnification with an inverted microscope. Eight hours later wells were reimaged and total migration distance was assessed according to gap closure. Blue: non-transduced; red: non-target; yellow: GIPC1 KD. (TIF) [file pone.0015581.s003.tif]
