# Supplementary material for: Therapeutic Implications of GIPC1 Silencing in Cancer
Source: PLoS One. 2010 Dec 30;5(12):e15581. doi: 10.1371/journal.pone.0015581 (PMC3012716; doi:10.1371/journal.pone.0015581)
Supplement: Table S1 — The set of the top 411 SAM probesets (0% FDR; fold change ≥ 2) assessed for clinical relevance to human breast and ovarian cancers with the globaltest Bioconductor R package. (DOC) [file pone.0015581.s004.doc]

**Table SI**

| Sample_ID | Expected score (dExp) | Observed score(d) | Numerator(r) | Denominator (s+s0) | Fold change | q-value (%) | GENE_SYMBOL |
| --- | --- | --- | --- | --- | --- | --- | --- |
| 223047_at | 0.12425086 | -11.891032 | -3.1849413 | 0.26784396 | 0.1146576 | 0 | CMTM6 |
| 217947_at | -0.007367376 | -19.586916 | -2.94144 | 0.15017372 | 0.1306717 | 0 | CMTM6 |
| 201890_at | -0.459991 | -17.824333 | -2.8840756 | 0.16180553 | 0.1360072 | 0 | RRM2 |
| 226847_at | 0.22653952 | 12.586624 | 2.5720398 | 0.20434707 | 5.9497886 | 0 | FST |
| 224850_at | 0.1717303 | -13.199724 | -2.555979 | 0.19363882 | 0.1689238 | 0 | ATAD1 |
| 212605_s_at | -0.14478984 | -11.904305 | -2.3922338 | 0.20095535 | 0.1906593 | 0 | NA |
| 221932_s_at | 0.09495332 | -14.365736 | -2.31844 | 0.16138679 | 0.201574 | 0 | GLRX5 |
| 203211_s_at | -0.41398942 | -11.415531 | -2.2911432 | 0.20070404 | 0.2046942 | 0 | MTMR2 |
| 225300_at | 0.18389587 | -12.846702 | -2.282185 | 0.17764755 | 0.2066841 | 0 | C15orf23 |
| 208652_at | -0.24923056 | -9.720282 | -2.18679 | 0.22497188 | 0.2233698 | 0 | PPP2CA |
| 204948_s_at | -0.35776833 | 13.726163 | 2.0937786 | 0.15253925 | 4.258029 | 0 | FST |
| 202499_s_at | -0.4384127 | 12.968323 | 2.0846767 | 0.16075145 | 4.232959 | 0 | SLC2A3 |
| 200777_s_at | -0.5011341 | -13.287986 | -2.0624905 | 0.15521468 | 0.2399119 | 0 | BZW1///LOC151579 |
| 212205_at | -0.15529704 | -10.336338 | -2.0639849 | 0.19968241 | 0.24164 | 0 | H2AFV |
| 209248_at | -0.23262377 | -13.05128 | -2.004971 | 0.15362255 | 0.2492062 | 0 | GHITM |
| 217845_x_at | -0.009975949 | -12.215605 | -2.0051837 | 0.16414936 | 0.249776 | 0 | HIGD1A |
| 229803_s_at | 0.31223056 | -16.008001 | -1.9860129 | 0.12406377 | 0.2523134 | 0 | NA |
| 216952_s_at | -0.032711532 | -13.636125 | -1.9556012 | 0.14341328 | 0.2579022 | 0 | LMNB2 |
| 227309_at | 0.23957367 | 7.6796885 | 1.9910486 | 0.25926164 | 3.8614736 | 0 | YOD1 |
| 212110_at | -0.15779263 | -12.466545 | -1.9395704 | 0.15558203 | 0.2611538 | 0 | SLC39A14 |
| 212608_s_at | -0.14470823 | -7.8855453 | -1.9577298 | 0.24826816 | 0.2630783 | 0 | NA |
| 203266_s_at | -0.41220286 | -9.000887 | -1.9242983 | 0.21378985 | 0.265525 | 0 | MAP2K4 |
| 221896_s_at | 0.09401776 | -12.084188 | -1.8820133 | 0.15574181 | 0.272056 | 0 | HIGD1A |
| 221791_s_at | 0.09127228 | -16.95655 | -1.8674803 | 0.11013327 | 0.2739818 | 0 | CCDC72 |
| 213154_s_at | -0.1304089 | -8.097136 | -1.845792 | 0.22795618 | 0.2759816 | 0 | BICD2 |
| 208078_s_at | -0.26513386 | 11.029538 | 1.8592639 | 0.16857132 | 3.6139665 | 0 | SNF1LK |
| 201721_s_at | -0.4661335 | -10.931875 | -1.8472748 | 0.1689806 | 0.2803965 | 0 | LAPTM5 |
| 209189_at | -0.23425078 | 11.477318 | 1.8208618 | 0.15864873 | 3.5126333 | 0 | FOS |
| 206924_at | -0.2978184 | -7.880152 | -1.8162639 | 0.23048589 | 0.2852823 | 0 | IL11 |
| 208787_at | -0.24549645 | -11.873316 | -1.8005991 | 0.1516509 | 0.2869093 | 0 | MRPL3 |
| 211976_at | -0.1613415 | -12.187439 | -1.7874327 | 0.14666188 | 0.2889099 | 0 | NA |
| 224641_at | 0.16614051 | -8.860901 | -1.78685 | 0.20165557 | 0.2930068 | 0 | FYTTD1 |
| 202833_s_at | -0.42684677 | -8.861444 | -1.7852781 | 0.20146582 | 0.2954403 | 0 | SERPINA1 |
| 203963_at | -0.3890918 | -8.743811 | -1.7515306 | 0.20031662 | 0.3012866 | 0 | CA12 |
| 200792_at | -0.50054425 | -14.615975 | -1.723793 | 0.117938966 | 0.3028833 | 0 | XRCC6 |
| 201343_at | -0.4799138 | -7.7504697 | -1.7325349 | 0.22353934 | 0.3056279 | 0 | UBE2D2 |
| 201150_s_at | -0.4870407 | -11.303322 | -1.7094374 | 0.15123318 | 0.305943 | 0 | TIMP3 |
| 208025_s_at | -0.26660028 | 9.484698 | 1.7103891 | 0.18033142 | 3.2415938 | 0 | HMGA2 |
| 219911_s_at | 0.04294947 | -12.208266 | -1.6952891 | 0.13886404 | 0.3094665 | 0 | SLCO4A1 |
| 209247_s_at | -0.23265353 | -10.741908 | -1.6891484 | 0.15724845 | 0.3097473 | 0 | ABCF2 |
| 200799_at | -0.50028104 | -9.031181 | -1.6865268 | 0.18674487 | 0.3116601 | 0 | HSPA1A |
| 234926_s_at | 0.48357597 | -12.573377 | -1.6797099 | 0.13359259 | 0.3125543 | 0 | C20orf43 |
| 213060_s_at | -0.13288184 | -8.3462515 | -1.6950378 | 0.20308971 | 0.3133062 | 0 | CHI3L2 |
| 212846_at | -0.1384352 | -8.932679 | -1.674747 | 0.18748541 | 0.3141023 | 0 | RRP1B |
| 202357_s_at | -0.44343832 | -10.137744 | -1.6821914 | 0.1659335 | 0.3144709 | 0 | CFB |
| 210665_at | -0.19457631 | 10.647094 | 1.6416535 | 0.15418795 | 3.1297355 | 0 | TFPI |
| 222088_s_at | 0.09904418 | 7.839954 | 1.6589837 | 0.21160631 | 3.1103227 | 0 | SLC2A14///SLC2A3 |
| 200065_s_at | -0.50956297 | -14.75861 | -1.629333 | 0.110398814 | 0.3230894 | 0 | ARF1 |
| 213599_at | -0.11884102 | -7.6845064 | -1.6623559 | 0.21632566 | 0.3237007 | 0 | OIP5 |
| 212206_s_at | -0.1552708 | -9.070543 | -1.6327696 | 0.18000792 | 0.3240428 | 0 | H2AFV |
| 213348_at | -0.12541327 | 9.1967535 | 1.6319976 | 0.17745367 | 3.080072 | 0 | CDKN1C |
| 234983_at | 0.4857435 | -8.669058 | -1.6069882 | 0.18537056 | 0.3250189 | 0 | NA |
| 230788_at | 0.34263584 | -9.747052 | -1.6258335 | 0.16680259 | 0.325408 | 0 | GCNT2 |
| 209112_at | -0.23644 | 8.500093 | 1.6313958 | 0.1919268 | 3.069235 | 0 | CDKN1B |
| 202811_at | -0.4276077 | -9.288517 | -1.6230454 | 0.17473677 | 0.3259336 | 0 | STAMBP |
| 200776_s_at | -0.5011892 | -10.677418 | -1.6072683 | 0.15052968 | 0.3286861 | 0 | BZW1///LOC151579 |
| 219534_x_at | 0.033251792 | 10.729187 | 1.5872269 | 0.14793542 | 3.0160258 | 0 | CDKN1C |
| 200977_s_at | -0.4935372 | -11.795465 | -1.5914598 | 0.13492131 | 0.332133 | 0 | TAX1BP1 |
| 200976_s_at | -0.49356937 | -11.489783 | -1.5824571 | 0.13772732 | 0.3343033 | 0 | TAX1BP1 |
| 226757_at | 0.2240251 | -8.746734 | -1.5953145 | 0.18238974 | 0.3343866 | 0 | IFIT2 |
| 212295_s_at | -0.15290667 | -9.731606 | -1.5782456 | 0.16217731 | 0.3343899 | 0 | SLC7A1 |
| 221699_s_at | 0.088877805 | -11.43662 | -1.5737982 | 0.13761044 | 0.3363304 | 0 | DDX50 |
| 224654_at | 0.16648062 | -10.405335 | -1.5702834 | 0.15091138 | 0.3371247 | 0 | DDX21 |
| 224604_at | 0.16512881 | -9.76006 | -1.5704737 | 0.16090819 | 0.3375927 | 0 | LOC401152 |
| 204774_at | -0.36324438 | 7.931046 | 1.5794258 | 0.1991447 | 2.9602556 | 0 | EVI2A |
| 217944_at | -0.007446473 | -10.26051 | -1.571331 | 0.15314355 | 0.3379893 | 0 | POMGNT1 |
| 201041_s_at | -0.49113196 | 12.956213 | 1.556314 | 0.120121054 | 2.9422157 | 0 | DUSP1 |
| 208750_s_at | -0.24655953 | -12.763706 | -1.5503273 | 0.12146372 | 0.341754 | 0 | ARF1 |
| 208152_s_at | -0.26308978 | -10.561228 | -1.5521364 | 0.14696553 | 0.3423751 | 0 | DDX21 |
| 222752_s_at | 0.11642941 | -7.4382524 | -1.57602 | 0.21188042 | 0.3423963 | 0 | C1orf75 |
| 202731_at | -0.43034434 | 9.236561 | 1.5283289 | 0.16546515 | 2.8834395 | 0 | PDCD4 |
| 204808_s_at | -0.3621707 | -10.032562 | -1.5229688 | 0.15180257 | 0.3488704 | 0 | TMEM5 |
| 204475_at | -0.37266082 | -12.028718 | -1.5169992 | 0.12611479 | 0.3490581 | 0 | MMP1 |
| 212298_at | -0.15282395 | -8.523657 | -1.531148 | 0.17963511 | 0.3490781 | 0 | NRP1 |
| 213738_s_at | -0.115204826 | -12.322253 | -1.5210152 | 0.12343645 | 0.3491453 | 0 | ATP5A1 |
| 223020_at | 0.12352664 | -9.77618 | -1.5177016 | 0.15524484 | 0.3504921 | 0 | CLPTM1L |
| 219330_at | 0.02802896 | -9.233051 | -1.5138116 | 0.16395572 | 0.3524522 | 0 | VANGL1 |
| 202859_x_at | -0.42595693 | -7.375968 | -1.5212812 | 0.20624836 | 0.3530418 | 0 | IL8 |
| 203632_s_at | -0.39991966 | 6.4154596 | 1.5134268 | 0.2359031 | 2.8149335 | 0 | GPRC5B |
| 226038_at | 0.20410328 | 6.8278213 | 1.4730308 | 0.2157395 | 2.8018172 | 0 | LONRF1 |
| 225856_at | 0.19914144 | -6.143922 | -1.4834912 | 0.24145672 | 0.3577787 | 0 | NA |
| 225232_at | 0.18208605 | -6.5190735 | -1.5109582 | 0.23177499 | 0.3590366 | 0 | MTMR12 |
| 208894_at | -0.24249993 | -10.734606 | -1.4742146 | 0.1373329 | 0.3605197 | 0 | HLA-DRA |
| 216250_s_at | -0.050648186 | -5.895859 | -1.4993904 | 0.25431246 | 0.360649 | 0 | LPXN |
| 200832_s_at | -0.4989985 | 9.829607 | 1.4706755 | 0.14961691 | 2.7664614 | 0 | SCD |
| 224623_at | 0.16563657 | -10.442347 | -1.4695892 | 0.14073361 | 0.3616784 | 0 | LOC728554///THOC3 |
| 233842_x_at | 0.44397283 | -11.405839 | -1.4674821 | 0.1286606 | 0.3624192 | 0 | C20orf43 |
| 207675_x_at | -0.2762044 | -7.5256834 | -1.4777069 | 0.19635518 | 0.3628255 | 0 | ARTN |
| 225036_at | 0.17673783 | -10.220629 | -1.465621 | 0.14339831 | 0.3629888 | 0 | C9orf105///SHB |
| 204731_at | -0.36462295 | 6.3488383 | 1.425628 | 0.22454941 | 2.7495174 | 0 | TGFBR3 |
| 213786_at | -0.11396605 | -5.7617626 | -1.4799609 | 0.25685906 | 0.3637989 | 0 | TAX1BP1 |
| 221253_s_at | 0.07737864 | -12.044529 | -1.4517021 | 0.12052793 | 0.3657407 | 0 | TXNDC5 |
| 224481_s_at | 0.161883 | -9.735687 | -1.4463348 | 0.14856012 | 0.3665864 | 0 | HECTD1 |
| 208638_at | -0.24963045 | -9.414686 | -1.4513893 | 0.15416226 | 0.3665954 | 0 | PDIA6 |
| 204830_x_at | -0.3614865 | 7.1625395 | 1.4675248 | 0.2048889 | 2.7266228 | 0 | PSG5 |
| 211429_s_at | -0.175362 | -11.152513 | -1.441659 | 0.12926763 | 0.3679076 | 0 | SERPINA1 |
| 205660_at | -0.33568716 | -11.538891 | -1.4417267 | 0.124945 | 0.3690235 | 0 | OASL |
| 217737_x_at | -0.012732201 | -10.869419 | -1.4384575 | 0.13233986 | 0.3699438 | 0 | C20orf43 |
| 212063_at | -0.15905336 | -10.1623745 | -1.4256225 | 0.14028439 | 0.3709596 | 0 | CD44 |
| 219032_x_at | 0.020374818 | 8.233094 | 1.4351344 | 0.17431289 | 2.6932464 | 0 | OPN3 |
| 202669_s_at | -0.4324615 | -8.946384 | -1.4336753 | 0.16025192 | 0.3714109 | 0 | EFNB2 |
| 214164_x_at | -0.104178056 | -6.97271 | -1.4527974 | 0.20835477 | 0.373003 | 0 | CA12 |
| 201288_at | -0.48192796 | -8.23237 | -1.4324789 | 0.17400564 | 0.3732652 | 0 | ARHGDIB |
| 233825_s_at | 0.44336927 | -9.849189 | -1.4269223 | 0.14487714 | 0.3734216 | 0 | CD99L2 |
| 201195_s_at | -0.48537642 | -11.867252 | -1.4229646 | 0.11990683 | 0.3734424 | 0 | SLC7A5 |
| 211708_s_at | -0.1681226 | 8.707462 | 1.4288025 | 0.16408943 | 2.673468 | 0 | SCD |
| 1554510_s_at | -1.0788268 | -9.025571 | -1.4269285 | 0.15809843 | 0.3743941 | 0 | GHITM |
| 222824_at | 0.118300974 | -6.523729 | -1.393476 | 0.21360116 | 0.374648 | 0 | NUDT5 |
| 216894_x_at | -0.034192156 | 9.06488 | 1.4085774 | 0.15538841 | 2.666012 | 0 | CDKN1C |
| 209312_x_at | -0.23083639 | -10.071785 | -1.4180312 | 0.14079244 | 0.3756552 | 0 | HLA-DRB1///LOC730415 |
| 209270_at | -0.23201491 | -8.417495 | -1.4114714 | 0.16768306 | 0.3768527 | 0 | LAMB3 |
| 220014_at | 0.045540757 | 7.254149 | 1.4020653 | 0.1932777 | 2.6528225 | 0 | PRR16 |
| 225381_at | 0.18612722 | 7.1056776 | 1.4018807 | 0.19729023 | 2.6439793 | 0 | LOC399959 |
| 208319_s_at | -0.2584216 | -8.2232 | -1.3982568 | 0.17003804 | 0.3793144 | 0 | RBM3 |
| 209630_s_at | -0.22215031 | -6.939233 | -1.3921468 | 0.2006197 | 0.3802044 | 0 | FBXW2 |
| 202054_s_at | -0.45411333 | -7.1407895 | -1.3998475 | 0.1960354 | 0.3805007 | 0 | ALDH3A2 |
| 238021_s_at | 0.615214 | -6.775536 | -1.4063289 | 0.2075598 | 0.3823834 | 0 | hCG_1815491 |
| 209163_at | -0.23500547 | -9.453365 | -1.3843203 | 0.14643677 | 0.3830624 | 0 | CYB561 |
| 228284_at | 0.2673556 | 8.313021 | 1.3804502 | 0.1660588 | 2.6090953 | 0 | TLE1 |
| 227531_at | 0.24583966 | -5.4732513 | -1.4306142 | 0.26138288 | 0.3845843 | 0 | NA |
| 202594_at | -0.43507314 | -7.6108084 | -1.3709483 | 0.18013176 | 0.3849946 | 0 | LEPROTL1 |
| 210982_s_at | -0.18650445 | -9.849278 | -1.3715415 | 0.13925299 | 0.3869618 | 0 | HLA-DRA |
| 225974_at | 0.20233533 | 7.0967197 | 1.3863096 | 0.19534513 | 2.5764785 | 0 | TMEM64 |
| 202946_s_at | -0.42297858 | 7.7012887 | 1.3656073 | 0.17732191 | 2.5700736 | 0 | BTBD3 |
| 206994_at | -0.2957589 | -11.621191 | -1.3601413 | 0.11703975 | 0.3893335 | 0 | CST4 |
| 206825_at | -0.30071494 | 9.753913 | 1.3590279 | 0.13933155 | 2.5678782 | 0 | OXTR |
| 206224_at | -0.31862646 | -11.043968 | -1.3602471 | 0.123166524 | 0.3895377 | 0 | CST1 |
| 209210_s_at | -0.23367237 | 7.2299824 | 1.3757606 | 0.19028546 | 2.5623999 | 0 | PLEKHC1 |
| 213183_s_at | -0.12967622 | 8.071889 | 1.3577149 | 0.16820288 | 2.5622911 | 0 | CDKN1C |
| 206059_at | -0.3235669 | 7.235913 | 1.3348708 | 0.18447857 | 2.5378575 | 0 | ZNF91 |
| 203983_at | -0.38844222 | -5.352716 | -1.367974 | 0.25556633 | 0.3942234 | 0 | TSNAX |
| 225492_at | 0.18917893 | -5.1858597 | -1.3667192 | 0.26354727 | 0.3948563 | 0 | NA |
| 209682_at | -0.22074148 | 6.067269 | 1.3378005 | 0.22049467 | 2.5311978 | 0 | CBLB |
| 227473_at | 0.2442729 | -10.0235195 | -1.342402 | 0.13392521 | 0.3951567 | 0 | NA |
| 44654_at | 1.505806 | -8.570979 | -1.3479614 | 0.15727042 | 0.3952925 | 0 | G6PC3 |
| 204435_at | -0.37392306 | 6.563768 | 1.336416 | 0.20360501 | 2.5270965 | 0 | NUPL1 |
| 224642_at | 0.16616748 | -6.0811777 | -1.3655577 | 0.2245548 | 0.3958641 | 0 | FYTTD1 |
| 226287_at | 0.21101229 | -5.2040243 | -1.3574419 | 0.26084465 | 0.3973447 | 0 | CCDC34 |
| 209249_s_at | -0.23259571 | -9.670848 | -1.3272586 | 0.13724326 | 0.3974578 | 0 | GHITM |
| 242775_at | 0.9467063 | -5.0407166 | -1.3423398 | 0.2662994 | 0.3978987 | 0 | NA |
| 223839_s_at | 0.14515275 | 6.722801 | 1.3412137 | 0.19950221 | 2.5117311 | 0 | SCD |
| 208607_s_at | -0.25046703 | -8.864056 | -1.3266711 | 0.14966863 | 0.3988435 | 0 | SAA1///SAA2 |
| 209433_s_at | -0.22752944 | -6.5785003 | -1.3373449 | 0.20329024 | 0.4007327 | 0 | PPAT |
| 225284_at | 0.18347296 | -8.264708 | -1.3207839 | 0.15981011 | 0.4011341 | 0 | DNAJC3///LOC144871 |
| 203394_s_at | -0.40787235 | 7.775601 | 1.3092222 | 0.1683757 | 2.492437 | 0 | HES1 |
| 201739_at | -0.4654761 | 8.215406 | 1.318284 | 0.16046485 | 2.485703 | 0 | SGK |
| 213182_x_at | -0.12969993 | 7.9348726 | 1.3031383 | 0.16422926 | 2.4794233 | 0 | CDKN1C |
| 1557100_s_at | -0.8702986 | -6.19287 | -1.2947466 | 0.20907053 | 0.4037494 | 0 | HECTD1 |
| 208713_at | -0.24758385 | -7.1471686 | -1.3107615 | 0.1833959 | 0.4052698 | 0 | HNRPUL1 |
| 225956_at | 0.20182258 | 6.1616616 | 1.29795 | 0.21064936 | 2.4654112 | 0 | LOC153222 |
| 204341_at | -0.37694708 | -8.982377 | -1.3048558 | 0.14526843 | 0.4056798 | 0 | TRIM16///TRIM16L |
| 212950_at | -0.13576898 | -8.2084055 | -1.3041477 | 0.15887955 | 0.405817 | 0 | GPR116 |
| 1553984_s_at | -1.1458123 | -8.565169 | -1.3047123 | 0.15232767 | 0.4062437 | 0 | DTYMK///LOC727761 |
| 241955_at | 0.8657771 | -5.5441585 | -1.2942369 | 0.23344155 | 0.4066051 | 0 | HECTD1 |
| 223076_s_at | 0.12501404 | -8.801782 | -1.3022122 | 0.14794871 | 0.407578 | 0 | NSUN2 |
| 201042_at | -0.49109852 | -7.9634814 | -1.3026581 | 0.16357897 | 0.4082819 | 0 | TGM2 |
| 202364_at | -0.44318748 | 7.491459 | 1.3020415 | 0.17380346 | 2.4487603 | 0 | MXI1 |
| 208999_at | -0.23957802 | -7.3106194 | -1.2943118 | 0.17704543 | 0.409625 | 0 | NA |
| 202297_s_at | -0.44551733 | -8.777429 | -1.2800417 | 0.14583333 | 0.4103442 | 0 | RER1 |
| 215193_x_at | -0.07776211 | -10.366866 | -1.2866168 | 0.12410856 | 0.4106325 | 0 | HLA-DRB1///HLA-DRB3 |
| 203323_at | -0.4102829 | 8.865126 | 1.2886243 | 0.14535883 | 2.434997 | 0 | CAV2 |
| 226625_at | 0.22035238 | 7.455138 | 1.2736335 | 0.17083968 | 2.4321547 | 0 | NA |
| 200673_at | -0.50514394 | -9.452238 | -1.2814307 | 0.13556902 | 0.4113681 | 0 | LAPTM4A |
| 224841_x_at | 0.17149548 | 10.413709 | 1.2780457 | 0.12272723 | 2.427522 | 0 | GAS5 |
| 224741_x_at | 0.16881669 | 11.165544 | 1.2789602 | 0.11454527 | 2.427477 | 0 | GAS5 |
| 242338_at | 0.9013898 | 5.485402 | 1.2717371 | 0.23184027 | 2.4240034 | 0 | TMEM64 |
| 223084_s_at | 0.12521927 | -7.3194036 | -1.2865481 | 0.17577226 | 0.4128971 | 0 | CCNDBP1 |
| 204602_at | -0.3686401 | 10.262383 | 1.2686529 | 0.123621665 | 2.4067793 | 0 | DKK1 |
| 208865_at | -0.24333715 | -8.8469305 | -1.263102 | 0.14277291 | 0.415771 | 0 | CSNK1A1 |
| 229334_at | 0.29819983 | 7.292524 | 1.2564819 | 0.17229725 | 2.4024832 | 0 | NA |
| 215867_x_at | -0.060443755 | -7.4241576 | -1.2750587 | 0.17174457 | 0.416635 | 0 | CA12 |
| 218647_s_at | 0.010516493 | -7.9171658 | -1.2702789 | 0.16044617 | 0.4168384 | 0 | YRDC |
| 224177_s_at | 0.15400018 | -8.0281725 | -1.2598872 | 0.15693325 | 0.4175938 | 0 | CXorf26 |
| 238022_at | 0.61526257 | -6.811764 | -1.2665782 | 0.18593983 | 0.4176405 | 0 | hCG_1815491 |
| 225579_at | 0.19154377 | 7.9196396 | 1.2623982 | 0.15940097 | 2.3942451 | 0 | PQLC3 |
| 242321_at | 0.8996547 | -4.6355166 | -1.2941625 | 0.2791841 | 0.4181302 | 0 | NA |
| 225645_at | 0.19335885 | -5.546381 | -1.256475 | 0.22653961 | 0.4182216 | 0 | EHF |
| 224894_at | 0.17292047 | -6.694218 | -1.2627854 | 0.18863823 | 0.4182511 | 0 | YAP1 |
| 229641_at | 0.30735123 | 6.149898 | 1.2701333 | 0.20652916 | 2.390773 | 0 | NA |
| 204508_s_at | -0.37160423 | -6.3652024 | -1.2843065 | 0.20176995 | 0.41844 | 0 | CA12 |
| 220486_x_at | 0.057667885 | -7.724202 | -1.2559109 | 0.16259426 | 0.419224 | 0 | TMEM164 |
| 211967_at | -0.16156551 | -8.709539 | -1.2538342 | 0.14396103 | 0.4194474 | 0 | TMEM123 |
| 200880_at | -0.49720365 | -8.510997 | -1.2521901 | 0.14712614 | 0.4195747 | 0 | DNAJA1 |
| 207622_s_at | -0.27773684 | -10.020237 | -1.2519503 | 0.12494218 | 0.4195935 | 0 | ABCF2 |
| 218943_s_at | 0.01809073 | -6.449168 | -1.2626982 | 0.1957924 | 0.42012 | 0 | DDX58 |
| 203821_at | -0.39372373 | -7.806627 | -1.257997 | 0.16114476 | 0.4203998 | 0 | HBEGF |
| 223474_at | 0.13545935 | -6.788377 | -1.2559657 | 0.1850171 | 0.4204612 | 0 | C14orf4 |
| 212959_s_at | -0.13553767 | 5.385737 | 1.2445369 | 0.23108014 | 2.3768313 | 0 | GNPTAB |
| 200954_at | -0.49438486 | -8.927934 | -1.2476182 | 0.13974322 | 0.4213614 | 0 | ATP6V0C |
| 207574_s_at | -0.27916613 | 8.967612 | 1.2495203 | 0.13933702 | 2.3724065 | 0 | GADD45B |
| 203302_at | -0.41099328 | -5.900255 | -1.266144 | 0.2145914 | 0.4217645 | 0 | DCK |
| 224763_at | 0.16936482 | 6.237695 | 1.2620337 | 0.20232372 | 2.3709855 | 0 | RPL37 |
| 219492_at | 0.03218608 | 7.576141 | 1.2518158 | 0.16523133 | 2.3699727 | 0 | CHIC2 |
| 213797_at | -0.11370486 | -6.1036 | -1.2608378 | 0.2065728 | 0.4224836 | 0 | RSAD2 |
| 203270_at | -0.41208258 | -8.118131 | -1.2500782 | 0.15398596 | 0.4225809 | 0 | DTYMK///LOC727761 |
| 204670_x_at | -0.3665528 | -9.384056 | -1.239656 | 0.13210236 | 0.4229238 | 0 | HLA-DRB1///LOC730415 |
| 214456_x_at | -0.096609026 | -11.104002 | -1.2419033 | 0.111842856 | 0.4229669 | 0 | SAA1 |
| 209209_s_at | -0.23369908 | 6.411227 | 1.2433958 | 0.19394037 | 2.3634772 | 0 | PLEKHC1 |
| 201906_s_at | -0.45940542 | -8.251254 | -1.2490659 | 0.15137891 | 0.4231566 | 0 | CTDSPL |
| 203925_at | -0.39032558 | -8.167799 | -1.2361641 | 0.15134604 | 0.4237136 | 0 | GCLM |
| 200783_s_at | -0.50089514 | -9.262562 | -1.2202196 | 0.13173673 | 0.4290039 | 0 | STMN1 |
| 201016_at | -0.49208516 | -5.5004783 | -1.2020874 | 0.21854234 | 0.4299433 | 0 | EIF1AX |
| 224480_s_at | 0.16185349 | -6.0675254 | -1.2346311 | 0.20348181 | 0.4304586 | 0 | MAG1 |
| 210797_s_at | -0.19116151 | -9.874094 | -1.2134523 | 0.12289252 | 0.4314555 | 0 | OASL |
| 224560_at | 0.16395345 | -7.823833 | -1.2086306 | 0.15448062 | 0.4318396 | 0 | TIMP2 |
| 36994_at | 1.4143542 | -11.418234 | -1.2101979 | 0.10598819 | 0.4323723 | 0 | ATP6V0C |
| 225955_at | 0.201798 | 8.197104 | 1.2096381 | 0.14756896 | 2.3088017 | 0 | LOC653506///METRNL |
| 225973_at | 0.20230599 | -7.102247 | -1.207768 | 0.17005435 | 0.4341413 | 0 | TAP2 |
| 209619_at | -0.22244674 | -10.27984 | -1.2029562 | 0.117020905 | 0.4341451 | 0 | CD74 |
| 1552309_a_at | -1.8317873 | 7.5105996 | 1.2057204 | 0.16053584 | 2.3009918 | 0 | NEXN |
| 217678_at | -0.014252955 | -6.1007833 | -1.2155721 | 0.19924854 | 0.434615 | 0 | SLC7A11 |
| 223041_at | 0.12408206 | -6.7166185 | -1.2122183 | 0.18048044 | 0.4362613 | 0 | CD99L2 |
| 210538_s_at | -0.19793902 | -5.242183 | -1.2142491 | 0.23163043 | 0.436426 | 0 | BIRC3 |
| 204020_at | -0.38726008 | -5.0880136 | -1.1931231 | 0.23449683 | 0.436844 | 0 | PURA |
| 202241_at | -0.4474487 | 6.8320827 | 1.2017057 | 0.17589156 | 2.2873888 | 0 | TRIB1 |
| 211003_x_at | -0.1859207 | -5.618422 | -1.2289062 | 0.21872799 | 0.4371984 | 0 | TGM2 |
| 225512_at | 0.18971132 | -4.8246408 | -1.2161677 | 0.25207424 | 0.4376892 | 0 | ZBTB38 |
| 222793_at | 0.11749518 | -5.60603 | -1.1964097 | 0.21341479 | 0.4379758 | 0 | DDX58 |
| 218205_s_at | -7.36E-04 | -7.087341 | -1.1929011 | 0.16831435 | 0.4390764 | 0 | MKNK2 |
| 1555812_a_at | -0.9522352 | -6.5500865 | -1.2027726 | 0.18362698 | 0.439175 | 0 | ARHGDIB |
| 225452_at | 0.18805675 | -7.130062 | -1.1822624 | 0.16581376 | 0.4395622 | 0 | MED1 |
| 202668_at | -0.43249512 | -5.6900544 | -1.1866941 | 0.20855585 | 0.4404463 | 0 | EFNB2 |
| 200831_s_at | -0.4990417 | 8.074321 | 1.178165 | 0.14591506 | 2.266678 | 0 | SCD |
| 226935_s_at | 0.22904436 | -8.014038 | -1.1835499 | 0.14768459 | 0.4411843 | 0 | CLPTM1L |
| 209213_at | -0.23358911 | -7.1968975 | -1.1862235 | 0.16482429 | 0.4413031 | 0 | CBR1 |
| 229441_at | 0.30135873 | 6.142875 | 1.1760044 | 0.19144201 | 2.265106 | 0 | PRSS23 |
| 213039_at | -0.1334428 | -8.376419 | -1.1820745 | 0.14111932 | 0.4418369 | 0 | ARHGEF18 |
| 231579_s_at | 0.36766246 | -7.9783397 | -1.1775322 | 0.14759113 | 0.4418425 | 0 | TIMP2 |
| 201132_at | -0.48772705 | -5.238343 | -1.2006037 | 0.22919534 | 0.4437689 | 0 | HNRPH2 |
| 211973_at | -0.16142099 | -5.7891927 | -1.170691 | 0.20222008 | 0.4440677 | 0 | NA |
| 209417_s_at | -0.2279525 | -7.2285094 | -1.1843939 | 0.16385037 | 0.4440754 | 0 | IFI35 |
| 1552310_at | -1.827816 | 5.0341787 | 1.1348498 | 0.22542898 | 2.2499723 | 0 | C15orf40 |
| 204885_s_at | -0.359741 | 7.142461 | 1.1718473 | 0.16406773 | 2.2489395 | 0 | MSLN |
| 202059_s_at | -0.45393834 | -5.873351 | -1.1581266 | 0.19718327 | 0.445134 | 0 | KPNA1 |
| 223294_at | 0.13067374 | -5.954889 | -1.186254 | 0.19920674 | 0.4451986 | 0 | CXorf26 |
| 211506_s_at | -0.17339054 | -6.6447196 | -1.1790347 | 0.17743935 | 0.4453091 | 0 | IL8 |
| 222735_at | 0.1159572 | -5.0773473 | -1.1779873 | 0.23200843 | 0.4453219 | 0 | TMEM38B |
| 1553976_a_at | -1.147022 | -6.540335 | -1.1708264 | 0.17901628 | 0.4453873 | 0 | RP11-529I10.4 |
| 216052_x_at | -0.055707604 | -7.7371864 | -1.167331 | 0.1508728 | 0.4457258 | 0 | ARTN |
| 207668_x_at | -0.2764186 | -8.824164 | -1.1622062 | 0.13170722 | 0.446174 | 0 | PDIA6 |
| 203871_at | -0.39209723 | -7.876067 | -1.1643662 | 0.147836 | 0.4462782 | 0 | SENP3 |
| 213640_s_at | -0.11776367 | 7.038721 | 1.1696043 | 0.16616715 | 2.2372253 | 0 | LOX |
| 217502_at | -0.018757533 | -6.723767 | -1.172235 | 0.174342 | 0.4471963 | 0 | IFIT2 |
| 219221_at | 0.025242258 | -7.5945225 | -1.1669097 | 0.15365149 | 0.4472024 | 0 | ZBTB38 |
| 205463_s_at | -0.34175435 | 5.867632 | 1.1743822 | 0.20014586 | 2.2338214 | 0 | PDGFA |
| 225210_s_at | 0.1814695 | -5.6335897 | -1.1636894 | 0.20656268 | 0.4478355 | 0 | FAM103A1 |
| 1554168_a_at | -1.1212825 | -8.982664 | -1.1584187 | 0.1289616 | 0.4480915 | 0 | SH3KBP1 |
| 212341_at | -0.15172473 | -7.8581777 | -1.1592693 | 0.14752394 | 0.4481143 | 0 | YIPF6 |
| 206052_s_at | -0.32375965 | -7.4965286 | -1.1627498 | 0.1551051 | 0.4481734 | 0 | SLBP |
| 243805_at | 1.0853126 | 7.4957104 | 1.1589737 | 0.15461826 | 2.2300985 | 0 | CCBE1 |
| 209675_s_at | -0.22094521 | -7.1987267 | -1.1529555 | 0.16016103 | 0.4491903 | 0 | HNRPUL1 |
| 201573_s_at | -0.47148028 | -7.0303955 | -1.1602354 | 0.16503131 | 0.4492375 | 0 | ETF1 |
| 202498_s_at | -0.43845215 | 6.8675857 | 1.1413984 | 0.16620083 | 2.2228935 | 0 | SLC2A3 |
| 202687_s_at | -0.43183208 | -7.4388623 | -1.160336 | 0.155983 | 0.4500068 | 0 | TNFSF10 |
| 202200_s_at | -0.4489198 | -8.782184 | -1.1514964 | 0.13111733 | 0.4500394 | 0 | SRPK1 |
| 202353_s_at | -0.44358918 | -4.782124 | -1.1715865 | 0.24499291 | 0.4508372 | 0 | PSMD12 |
| 216640_s_at | -0.040759586 | -9.945032 | -1.1490979 | 0.115544915 | 0.4511078 | 0 | PDIA6 |
| 202433_at | -0.44079128 | -8.54689 | -1.1511369 | 0.13468488 | 0.4513092 | 0 | SLC35B1 |
| 223244_s_at | 0.12933001 | -10.134929 | -1.1458321 | 0.11305773 | 0.4521326 | 0 | NDUFA12 |
| 226860_at | 0.22690262 | -5.2577014 | -1.1251206 | 0.21399477 | 0.4530912 | 0 | TMEM19 |
| 223009_at | 0.12324009 | -10.525829 | -1.1420293 | 0.1084978 | 0.4531908 | 0 | C11orf59 |
| 216236_s_at | -0.051004004 | 6.9210896 | 1.149291 | 0.16605636 | 2.2056763 | 0 | SLC2A14///SLC2A3 |
| 235004_at | 0.48654398 | 4.579199 | 1.1135302 | 0.2431714 | 2.2053003 | 0 | RBM24 |
| 1555790_a_at | -0.9537784 | 5.7510505 | 1.1315308 | 0.19675201 | 2.2006817 | 0 | FLJ38482///ZNF320 |
| 225120_at | 0.17900878 | -4.488355 | -1.173563 | 0.26146838 | 0.4547908 | 0 | PURB |
| 212531_at | -0.14676322 | -8.826872 | -1.13729 | 0.12884405 | 0.4548313 | 0 | LCN2 |
| 223082_at | 0.12516645 | -8.351824 | -1.1340036 | 0.13577916 | 0.4550883 | 0 | SH3KBP1 |
| 227586_at | 0.24737352 | 6.029084 | 1.1462009 | 0.19011194 | 2.1959126 | 0 | TMEM170 |
| 225904_at | 0.20042802 | -6.23232 | -1.1325204 | 0.18171732 | 0.45582 | 0 | C1orf96 |
| 201117_s_at | -0.48827222 | 6.1062717 | 1.1460357 | 0.18768173 | 2.1926334 | 0 | CPE |
| 221759_at | 0.090429634 | -7.2845187 | -1.1377053 | 0.15618126 | 0.4561685 | 0 | G6PC3 |
| 218739_at | 0.012885978 | 7.520254 | 1.1235003 | 0.1493966 | 2.1821895 | 0 | ABHD5 |
| 225976_at | 0.20238754 | -7.656297 | -1.12503 | 0.1469418 | 0.458464 | 0 | BTF3L4 |
| 225847_at | 0.19890535 | -5.4328747 | -1.1218972 | 0.20650159 | 0.4586024 | 0 | AADACL1 |
| 200890_s_at | -0.4968236 | -5.253558 | -1.1304665 | 0.21518111 | 0.4587676 | 0 | SSR1 |
| 225099_at | 0.17844218 | -7.2221594 | -1.1291213 | 0.15634124 | 0.4594244 | 0 | FBXO45 |
| 219972_s_at | 0.044482034 | -4.7629395 | -1.1245322 | 0.23610047 | 0.4600064 | 0 | C14orf135 |
| 208639_x_at | -0.24960083 | -10.168668 | -1.1175008 | 0.10989648 | 0.4605952 | 0 | PDIA6 |
| 211020_at | -0.18547527 | -5.718088 | -1.1330402 | 0.19815017 | 0.4606574 | 0 | GCNT2 |
| 213037_x_at | -0.13349429 | -8.154734 | -1.118473 | 0.1371563 | 0.4606982 | 0 | STAU1 |
| 224767_at | 0.16947457 | 8.130601 | 1.1190162 | 0.1376302 | 2.1705787 | 0 | RPL37 |
| 208940_at | -0.24123234 | -5.2469845 | -1.1119909 | 0.21192953 | 0.4611509 | 0 | SEPHS1 |
| 202688_at | -0.4318027 | -7.152233 | -1.1208129 | 0.1567081 | 0.4611772 | 0 | TNFSF10 |
| 204298_s_at | -0.3783322 | 7.751159 | 1.1158743 | 0.14396225 | 2.1672864 | 0 | LOX |
| 202766_s_at | -0.429125 | 4.9206905 | 1.0929618 | 0.22211553 | 2.1627512 | 0 | FBN1 |
| 202936_s_at | -0.42331302 | -7.2183986 | -1.1221681 | 0.15545942 | 0.4624413 | 0 | SOX9 |
| 221858_at | 0.09301696 | 5.5262036 | 1.1065817 | 0.20024265 | 2.1605148 | 0 | TBC1D12 |
| 202589_at | -0.4352572 | -9.171869 | -1.1122704 | 0.121269755 | 0.463164 | 0 | TYMS |
| 202497_x_at | -0.43848404 | 7.831904 | 1.1115432 | 0.14192502 | 2.1533747 | 0 | SLC2A3 |
| 207243_s_at | -0.2885809 | -10.238004 | -1.1063032 | 0.10805849 | 0.4644388 | 0 | CALM2 |
| 201572_x_at | -0.47152102 | -6.1881585 | -1.1181221 | 0.18068737 | 0.4648408 | 0 | DCTD |
| 211509_s_at | -0.1733111 | -9.069791 | -1.1043301 | 0.121759154 | 0.4648785 | 0 | RTN4 |
| 209344_at | -0.22999857 | -8.025599 | -1.0977125 | 0.1367764 | 0.466779 | 0 | TPM4 |
| 229830_at | 0.31303954 | 6.00958 | 1.1106806 | 0.18481833 | 2.1420023 | 0 | NA |
| 228450_at | 0.272127 | 6.0867834 | 1.1003175 | 0.18077159 | 2.13998 | 0 | PLEKHA7 |
| 212070_at | -0.15886663 | -5.5734386 | -1.1205735 | 0.20105605 | 0.4673686 | 0 | GPR56 |
| 217388_s_at | -0.021695623 | -5.542633 | -1.0982323 | 0.19814269 | 0.4676296 | 0 | KYNU |
| 202053_s_at | -0.45414826 | -6.941821 | -1.0960398 | 0.15788937 | 0.4676686 | 0 | ALDH3A2 |
| 212702_s_at | -0.14226404 | -5.6903925 | -1.1170473 | 0.19630408 | 0.468113 | 0 | BICD2 |
| 222000_at | 0.09670339 | -5.3720827 | -1.0973577 | 0.20427045 | 0.4681626 | 0 | C1orf174 |
| 226604_at | 0.21975979 | -5.7976847 | -1.0834632 | 0.1868786 | 0.468575 | 0 | TMTC3 |
| 228910_at | 0.2856072 | -7.546237 | -1.1001024 | 0.14578159 | 0.4686037 | 0 | NA |
| 212951_at | -0.13574208 | -7.2853446 | -1.0988855 | 0.15083508 | 0.4687076 | 0 | GPR116 |
| 202634_at | -0.43371463 | -6.964282 | -1.0823522 | 0.15541475 | 0.4695091 | 0 | POLR2K |
| 224895_at | 0.17294313 | -7.4193416 | -1.0923119 | 0.1472249 | 0.4696485 | 0 | YAP1 |
| 39248_at | 1.4530236 | -7.396217 | -1.0944865 | 0.14797923 | 0.4697702 | 0 | AQP3 |
| 203395_s_at | -0.40783393 | 7.198215 | 1.0875831 | 0.15109067 | 2.128139 | 0 | HES1 |
| 205483_s_at | -0.34112096 | -9.128281 | -1.0902672 | 0.119438395 | 0.4699515 | 0 | ISG15 |
| 212593_s_at | -0.14510462 | 6.924228 | 1.096664 | 0.15838067 | 2.1267493 | 0 | PDCD4 |
| 205661_s_at | -0.33565515 | -6.460329 | -1.09184 | 0.16900687 | 0.470761 | 0 | FLAD1 |
| 207808_s_at | -0.27242646 | 5.8935204 | 1.0898538 | 0.18492407 | 2.1222477 | 0 | PROS1 |
| 227095_at | 0.23357122 | 4.2892623 | 1.0673838 | 0.2488502 | 2.1217256 | 0 | LEPROT |
| 218772_x_at | 0.013735603 | -4.2176404 | -1.1259205 | 0.26695508 | 0.4714545 | 0 | TMEM38B |
| 201439_at | -0.47633028 | -6.133952 | -1.0978713 | 0.1789827 | 0.4716013 | 0 | GBF1 |
| 203625_x_at | -0.40014333 | 7.187941 | 1.0891652 | 0.15152673 | 2.1202948 | 0 | SKP2 |
| 222006_at | 0.0968688 | -5.4614296 | -1.0984731 | 0.20113288 | 0.4717494 | 0 | LETM1 |
| 210095_s_at | -0.20960553 | -8.103263 | -1.0786853 | 0.1331174 | 0.4718362 | 0 | IGFBP3 |
| 218853_s_at | 0.015806157 | 4.5611224 | 1.0998578 | 0.24113753 | 2.1182613 | 0 | MOSPD1 |
| 203565_s_at | -0.40214834 | -8.770676 | -1.0861335 | 0.12383692 | 0.472112 | 0 | MNAT1 |
| 211653_x_at | -0.16951525 | -8.175282 | -1.0822816 | 0.13238463 | 0.4723863 | 0 | AKR1C2 |
| 224582_s_at | 0.16454527 | -7.2985716 | -1.0874753 | 0.14899838 | 0.4726094 | 0 | NUCKS1 |
| 212290_at | -0.15304266 | -6.0095725 | -1.0839453 | 0.18036978 | 0.4726583 | 0 | SLC7A1 |
| 228531_at | 0.27446696 | -5.11268 | -1.0788219 | 0.21100909 | 0.4728035 | 0 | SAMD9 |
| 209157_at | -0.23517369 | -4.984537 | -1.0927558 | 0.21922915 | 0.4730582 | 0 | DNAJA2 |
| 218528_s_at | 0.007460537 | 5.5846205 | 1.0716448 | 0.19189215 | 2.1136322 | 0 | RNF38 |
| 202149_at | -0.4507156 | -4.478084 | -1.0629046 | 0.237357 | 0.474851 | 0 | NEDD9 |
| 224233_s_at | 0.15542942 | -5.503527 | -1.0795264 | 0.19615173 | 0.4751984 | 0 | LOC731059///MSTO1 |
| 202915_s_at | -0.4240248 | -6.7547092 | -1.071393 | 0.15861423 | 0.4756839 | 0 | FAM20B |
| 1554696_s_at | -1.0579463 | -8.10824 | -1.0700512 | 0.13197084 | 0.4760085 | 0 | TYMS |
| 204194_at | -0.3816674 | 5.4593678 | 1.0723095 | 0.19641642 | 2.100482 | 0 | BACH1 |
| 218260_at | 6.69E-04 | -6.6465364 | -1.0736561 | 0.16153617 | 0.4761208 | 0 | C19orf58 |
| 201720_s_at | -0.46616843 | -6.1437607 | -1.076971 | 0.17529508 | 0.4761559 | 0 | LAPTM5 |
| 209921_at | -0.21425456 | -5.320413 | -1.0622964 | 0.19966426 | 0.4766137 | 0 | SLC7A11 |
| 201859_at | -0.46110272 | -5.6072707 | -1.0644712 | 0.18983768 | 0.476682 | 0 | SRGN |
| 225633_at | 0.19302785 | 5.808637 | 1.0585816 | 0.18224268 | 2.0950043 | 0 | DPY19L3 |
| 201428_at | -0.47673663 | -5.726838 | -1.0807128 | 0.1887102 | 0.4773914 | 0 | CLDN4 |
| 203447_at | -0.4061234 | -8.0332575 | -1.0670743 | 0.13283208 | 0.4782455 | 0 | PSMD5 |
| 217778_at | -0.011667047 | -6.2175603 | -1.0614333 | 0.1707154 | 0.4785442 | 0 | SLC39A1 |
| 225803_at | 0.197639 | 8.36819 | 1.0608802 | 0.12677535 | 2.0885022 | 0 | FBXO32 |
| 221751_at | 0.09021752 | -4.9205785 | -1.043972 | 0.21216449 | 0.4796979 | 0 | SLC2A3P1 |
| 217908_s_at | -0.008358141 | -5.687291 | -1.0765309 | 0.18928711 | 0.4797441 | 0 | IQWD1 |
| 225788_at | 0.19722658 | -9.729137 | -1.0578275 | 0.108727776 | 0.4800898 | 0 | C6orf153 |
| 210735_s_at | -0.19280271 | -5.7094007 | -1.0724063 | 0.18783167 | 0.4802034 | 0 | CA12 |
| 204268_at | -0.37932026 | -7.2025456 | -1.0553904 | 0.14653018 | 0.4821124 | 0 | S100A2 |
| 225485_at | 0.18898483 | -4.8224325 | -1.0665834 | 0.22117124 | 0.4822987 | 0 | TSGA14 |
| 212621_at | -0.1443657 | -5.150122 | -1.042131 | 0.20235072 | 0.4830055 | 0 | KIAA0286 |
| 221677_s_at | 0.08832375 | -5.3550544 | -1.0573072 | 0.197441 | 0.4832647 | 0 | DONSON |
| 242005_at | 0.87027717 | -4.4304113 | -1.0545475 | 0.23802476 | 0.484263 | 0 | NA |
| 223484_at | 0.13572764 | -6.081125 | -1.0472431 | 0.17221208 | 0.4848253 | 0 | C15orf48 |
| 202808_at | -0.42769977 | -6.6354275 | -1.0382781 | 0.15647493 | 0.4848812 | 0 | C10orf26 |
| 226155_at | 0.20734116 | 5.2977815 | 1.0351601 | 0.19539501 | 2.061278 | 0 | KIAA1600 |
| 212474_at | -0.14824495 | -4.392372 | -1.0325978 | 0.23508887 | 0.4856705 | 0 | KIAA0241 |
| 208079_s_at | -0.2651018 | -8.182498 | -1.0372763 | 0.12676768 | 0.4866648 | 0 | AURKA |
| 214697_s_at | -0.09046893 | 4.597473 | 1.0438027 | 0.22703835 | 2.052942 | 0 | ROD1 |
| 212097_at | -0.15813164 | -8.226437 | -1.0354443 | 0.12586789 | 0.4873714 | 0 | CAV1 |
| 210137_s_at | -0.20846127 | -6.2108173 | -1.0422359 | 0.16780977 | 0.487555 | 0 | DCTD |
| 206074_s_at | -0.32309303 | -8.608084 | -1.0339622 | 0.120115265 | 0.4877904 | 0 | HMGA1 |
| 212406_s_at | -0.15000996 | 4.6266074 | 1.0463037 | 0.22614925 | 2.0498888 | 0 | PCMTD2 |
| 208306_x_at | -0.25877613 | -7.220079 | -1.0376272 | 0.14371412 | 0.4880087 | 0 | HLA-DRB1 |
| 202677_at | -0.43218815 | -5.502763 | -1.0265617 | 0.18655388 | 0.4885358 | 0 | RASA1 |
| 218187_s_at | -0.001210938 | -5.4656653 | -1.0433002 | 0.19088255 | 0.4886987 | 0 | C8orf33 |
| 225665_at | 0.19389784 | -6.0416255 | -1.0397272 | 0.17209396 | 0.4887535 | 0 | ZAK |
| 223297_at | 0.13075331 | -4.956696 | -1.0221553 | 0.20621707 | 0.488787 | 0 | AMMECR1L |
| 201531_at | -0.47297645 | 8.580137 | 1.0325279 | 0.12033933 | 2.0442514 | 0 | ZFP36 |
| 223132_s_at | 0.12642772 | -8.076403 | -1.0313029 | 0.12769336 | 0.4892052 | 0 | TRIM8 |
| 226140_s_at | 0.20691948 | 6.929192 | 1.0345821 | 0.14930776 | 2.0434508 | 0 | OTUD1 |
| 223828_s_at | 0.14486803 | -5.9571967 | -1.027385 | 0.17246115 | 0.4895675 | 0 | LGALS12 |
| 218754_at | 0.013264708 | -5.487711 | -1.04565 | 0.19054393 | 0.4899918 | 0 | NOL9 |
| 218571_s_at | 0.008584213 | -5.247511 | -1.042192 | 0.19860692 | 0.4902357 | 0 | CHMP4A |
| 202466_at | -0.43958554 | -7.2502627 | -1.0248837 | 0.14135815 | 0.4906282 | 0 | POLS |
| 202593_s_at | -0.4351175 | -5.4071636 | -1.0404968 | 0.19242932 | 0.4907735 | 0 | MIR16 |
| 212886_at | -0.13738805 | -4.818995 | -1.0613642 | 0.22024596 | 0.4908701 | 0 | CCDC69 |
| 212482_at | -0.14803405 | 4.981164 | 1.020272 | 0.20482603 | 2.0320916 | 0 | RMND5A |
| 211162_x_at | -0.1817151 | 4.997573 | 1.0561056 | 0.21132371 | 2.0317793 | 0 | SCD |
| 222557_at | 0.11128442 | -6.0799513 | -1.0320783 | 0.16975108 | 0.4925581 | 0 | STMN3 |
| 225450_at | 0.1880065 | -6.394095 | -1.0269222 | 0.16060477 | 0.4926055 | 0 | AMOTL1 |
| 222453_at | 0.108531795 | 5.4106183 | 1.0157418 | 0.18773119 | 2.0282578 | 0 | CYBRD1 |
| 213397_x_at | -0.124127984 | 5.303925 | 1.0276451 | 0.19375181 | 2.0262978 | 0 | RNASE4 |
| 212731_at | -0.14148389 | 4.9307947 | 1.0055213 | 0.20392682 | 2.026271 | 0 | ANKRD46 |
| 226302_at | 0.21141727 | 3.9974916 | 1.0006351 | 0.25031576 | 2.0250082 | 0 | ATP8B1 |
| 227503_at | 0.24507558 | 4.602228 | 0.9913144 | 0.2153988 | 2.0244033 | 0 | NA |
| 202023_at | -0.4551986 | 5.487411 | 1.0322566 | 0.1881136 | 2.0239327 | 0 | EFNA1 |
| 224871_at | 0.17230247 | 7.58296 | 1.0158377 | 0.13396321 | 2.0239198 | 0 | FAM79A |
| 1554241_at | -1.1120844 | -6.3462877 | -1.0274048 | 0.16189067 | 0.4941299 | 0 | COCH |
| 227533_at | 0.24590103 | 4.4095616 | 1.034769 | 0.23466484 | 2.0227704 | 0 | NA |
| 225698_at | 0.19479124 | 7.6677117 | 1.0134759 | 0.13217449 | 2.0221283 | 0 | C5orf26 |
| 205571_at | -0.3383931 | 4.832423 | 1.022268 | 0.21154357 | 2.0216596 | 0 | LIPT1 |
| 201468_s_at | -0.47529015 | -8.355948 | -1.0159178 | 0.12158019 | 0.4946457 | 0 | NQO1 |
| 204070_at | -0.38562986 | -6.9480634 | -1.0200157 | 0.14680576 | 0.4948177 | 0 | RARRES3 |
| 203758_at | -0.39581662 | 6.1289277 | 1.0221624 | 0.16677672 | 2.020718 | 0 | CTSO |
| 207345_at | -0.28568056 | 5.8766766 | 1.0220888 | 0.17392293 | 2.0197685 | 0 | FST |
| 200931_s_at | -0.49525726 | -7.4140697 | -1.0137701 | 0.13673599 | 0.4952878 | 0 | VCL |
| 225205_at | 0.18133157 | -7.530868 | -1.0114741 | 0.13431042 | 0.4959408 | 0 | KIF3B |
| 210968_s_at | -0.186877 | -7.6508503 | -1.0099869 | 0.13200976 | 0.4963574 | 0 | RTN4 |
| 221012_s_at | 0.07117062 | -6.219808 | -1.0203538 | 0.16404907 | 0.4963712 | 0 | TRIM8 |
| 1558080_s_at | -0.82141995 | -4.5454345 | -1.0048149 | 0.22106025 | 0.4966749 | 0 | DNAJC3///LOC144871 |
| 226600_at | 0.21964917 | -4.0856967 | -1.0128622 | 0.2479044 | 0.4969718 | 0 | TMTC3 |
| 200891_s_at | -0.49678814 | -6.4752445 | -1.0052791 | 0.1552496 | 0.4972018 | 0 | SSR1 |
| 204698_at | -0.3656623 | -4.9968734 | -1.0141602 | 0.20295894 | 0.4973963 | 0 | ISG20 |
| 202478_at | -0.43915975 | -4.981809 | -1.0344782 | 0.20765111 | 0.4975152 | 0 | TRIB2 |
| 218681_s_at | 0.011413749 | -4.485047 | -1.0462203 | 0.23326853 | 0.4979977 | 0 | SDF2L1 |
| 228222_at | 0.26555103 | -6.510141 | -1.0072241 | 0.15471618 | 0.4980564 | 0 | PPP1CB |
| 211946_s_at | -0.16210866 | -5.018499 | -1.0232487 | 0.20389536 | 0.4982729 | 0 | BAT2D1 |
| 225935_at | 0.20129056 | -4.2145987 | -1.0247166 | 0.24313504 | 0.4985237 | 0 | NA |
| 226179_at | 0.20801438 | -5.597557 | -1.0150189 | 0.18133248 | 0.4986013 | 0 | SLC25A37 |
| 212680_x_at | -0.14283542 | -8.535902 | -1.0037332 | 0.117589585 | 0.4987085 | 0 | PPP1R14B |
| 209434_s_at | -0.22749862 | -4.9397163 | -1.0269794 | 0.20790252 | 0.4992183 | 0 | PPAT |
| 213179_at | -0.12977767 | -6.2358913 | -1.005033 | 0.1611691 | 0.4995042 | 0 | RQCD1 |
| 214755_at | -0.08898058 | 7.055892 | 1.0025597 | 0.1420883 | 2.0014825 | 0 | UAP1L1 |
| 215113_s_at | -0.079839185 | -5.1673927 | -0.99734783 | 0.19300795 | 0.4996473 | 0 | SENP3 |
| 214629_x_at | -0.09215142 | -7.770623 | -1.0015478 | 0.128889 | 0.4999663 | 0 | RTN4 |
| **Table SI.** The set of the top 411 SAM probesets (0% FDR; fold change ≥ 2) assessed for clinical relevance to human breast and ovarian cancers with the globaltest Bioconductor R package. | | | | | | | |
